# Influence of the nutritional status on facial morphology in young Japanese women

**DOI:** 10.1038/s41598-022-21919-5

**Published:** 2022-11-03

**Authors:** Chihiro Tanikawa, Miki Kurata, Noriko Tanizaki, Mika Takeuchi, Edlira Zere, Keisuke Fukuo, Kenji Takada

**Affiliations:** 1 Department of Orthodontics and Dentofacial Orthopedics, Osaka University Dental Hospital, Suita, Osaka Japan; 2 Center for Advanced Medical Engineering and Informatics, Osaka University, Suita, Osaka Japan; 3 Department of Food Sciences and Nutrition, School of Human Environmental Sciences, Mukogawa Women’s University, Nishinomiya, Hyogo Japan

**Keywords:** Nutrition, Body mass index, Craniofacial orthodontics

## Abstract

Evidence regarding the possible influence of nutritional status on the facial morphology has thus far been insufficient. We examined whether or not the physical body compositions and dietary behaviors were correlated with any morphological characteristics of the face. One hundred and fifteen young Japanese women participated. Variables representing the dietary behaviors were extracted from self-reported survey data, and corresponding three-dimensional (3D) facial images and body compositions were examined. Multivariate analyses identified significant relationships between the nutritional status and facial topography (p < 0.05). The clustering method revealed the existence of three dietary condition patterns (“balanced diet”, “high-calorie-diet” with obesity tendency, and “imbalanced low-calorie-diet” with sarcopenic obesity tendency). Among these three patterns, a round face (increased facial width; analysis of variance [ANOVA], p < 0.05) was observed in the high-calorie-diet pattern, while the imbalanced low-calorie-diet pattern showed a more masculine face (increased face height, decreased eye height, increased non-allometric sexual shape differences; ANOVA, p < 0.05), thus suggesting the possibility of sex-hormonal influences. In summary, the body composition and dietary behaviors were found to influence the facial morphology, and potential biological influences were discussed.

## Introduction

The morphology of the human face is influenced by both genetic and environmental factors and their complex interactions. Because the face is a crucial interface for social interactions, elucidating these effects on facial morphology has fascinated several disciplines, such as evolutional sciences, biology, forensics, and orthodontics^1,2^. Thus far, the decomposition of facial characteristics into genetic and environmental factors has been achieved by estimating heritability (e.g. classically estimated by the proportion of the total phenotypic variance explained by additive genetic variance^3,4^ and recently estimated by population-based genome-wide association studies^5,6^) with twin models and parent–offspring models. As the estimated heritability for the facial features mentioned above has been generally reported to range from 40 to 80%^7^, the remaining 20–60% of variance in facial morphology seems to be related to environmental factors and the interaction between environmental factors and heritability. However, previous studies have mainly focused on genetics, and evidence concerning the influence of the environment has been limited.

A number of environmental factors can affect facial characteristics, including hormones, nutrition, diseases, trauma, surgery, dentofacial orthopedics, lifestyle factors (smoking, drinking, exercise, etc.), and the oral function (chewing, breathing, and swallowing)^8^. Among these, the nutritional status is one of the most important factors affecting the body and even the facial morphology. Under adverse nutritional conditions (e.g. lack of dietary protein), the physical body height growth of children can decline, and even their adult height can be affected^9^. Regarding facial characteristics, many studies^10–12^ have shown subjects with a high body fat proportion to have facial characteristics of adiposity. A previous study^13^ indicated that subjects with increased buccal fat were more likely to have increased visceral fat, so patients with chubby cheeks may be at a higher risk of metabolic complications of obesity than others.

Furthermore, several computer algorithms have been proposed for automatically predicting obesity or the body mass index (BMI) using facial two-dimensional (2D) photographs^14,15^. A study using geometric morphometric modeling of facial landmark data in 2D photographs^16^ successfully predicted the BMI (32% of variance explained) and percentage body fat (21%). In addition to effects on soft tissue, recent studies have further clarified the influence of obesity or a high BMI on the hard-tissue craniofacial morphology using roentgen cephalograms^17^. These studies showed that obesity results in a greater facial size and bimaxillary protrusion with advanced maturation with or without vertical differences. This is assumed to be related to the fact that obesity can cause precocious puberty (earlier sexually development) and advanced bone maturity^17^, indicating the sex-hormonal association of obesity.

Sex hormones are another important factor related to facial shapes^18^. For example, several previous studies have shown that a high testosterone level in women was related to perceived ‘maleness’ in the face (a greater height-to-width ratio of the mid-face)^18^. A twin study using magnetic resonance imaging showed that women with a male twin, who were presumably exposed to greater fetal testosterone levels than most female neonates, exhibited greater facial ‘maleness’ characteristics than those with a female twin^19^. There have been several studies examining the relationships between eating behaviors and sex hormones^20–23^. For example, positive associations of saturated fat^21^ and total fat^20^ with estrogen concentrations have been observed in cross-sectional studies of premenopausal Japanese women^22^. A previous study clarified three distinct dietary patterns of "traditional diet," "unhealthy diet," and "protein diet," showing that the "unhealthy diet" pattern was significantly positively associated with precocious puberty in both boys and girls^23^. This suggests that the hormonal status is affected by the dietary intake and may be related to facial characteristics. It is therefore reasonable to consider that the dietary intake can influence the facial shape through sex hormones.

However, little is known at present regarding the influences of holistic nutritional status, including the dietary intake, eating behaviors, and body composition, on the facial form^24^. Furthermore, most of these previous findings were primarily based on 2D photos^17,25^, which leads to difficulties in discussion from a biological aspect. In addition, indices to express ‘maleness’ in previous studies^18,19^ were only based on observers’ perception; however, one study^26^ showed that the shape features perceived as masculine only partly resembled the biological facial sexual dimorphism characteristics.

Recent technological advances have shown that three-dimensional (3D) soft-tissue facial examinations using geometric morphometrics may provide useful information concerning the face^27,28^. For example, a previous study^29^ showed that the 3D facial features are more reliable aging biomarkers than blood profiles and can reflect the general health status better than chronological age. Another 3D study^30^ demonstrated a method to measure biological sexual shape dimorphic (SShD^31^) characteristics based on the 3D facial images obtained using an approach associated with morphometrics^31^. Non-allometric (i.e. non-size-related and shape-related) part of SShD sexual dimorphism has been considered to more closely reflect the effects of androgens than the allometric (i.e. size-related) aspects^26^. Thus, 3D facial images are assumed to be beneficial for examining the influences of eating behaviors including consequent sex hormonal changes on the facial morphology.

Therefore, the present study examined whether or not the nutritional status correlated with the 3D facial morphology in young Japanese women and discussed potential biological explanations for the relationship between facial characteristics and the nutritional status.

## Results

### Direct comparisons between nutritional status and 3D facial shapes

#### Pre-process of dietary intakes

**Figure 1:**
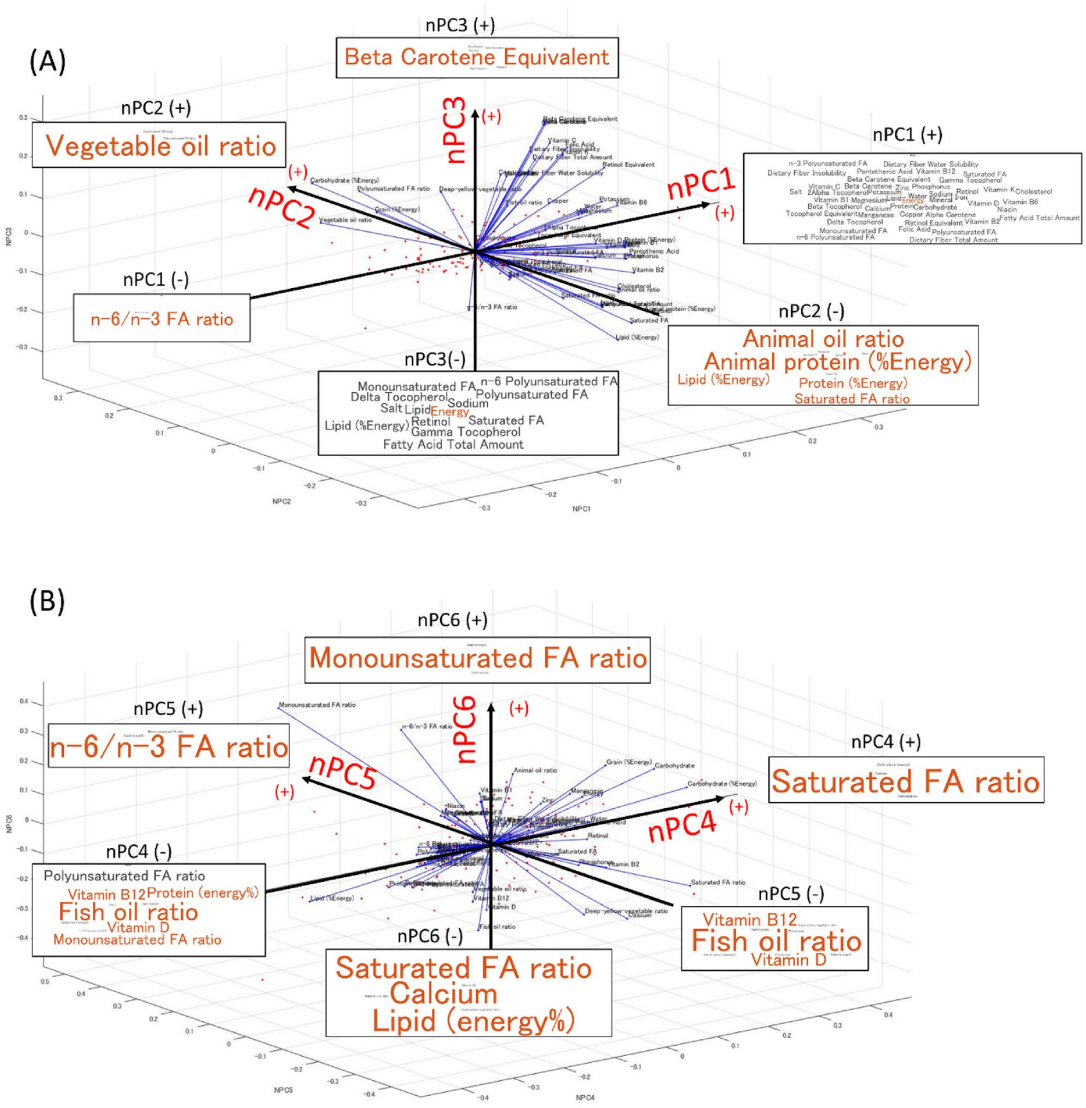
Plot of the participants (red dots) in the space of the nutritional principal components (nPCs). nPCs 1, 2, 3, 4, 5, 6, and 7 accounted for 52%, 10%, 9%, 7%, 6%, 4%, and 2% of the total variation in the samples, respectively. Word clouds at the ends of each axis indicate the direction of nPCs 1 through 6, where the relatively large and orange words are the most highly weighted words in each axis, while the smaller and black words were the secondary weighted words.

Based on a food frequency questionnaire collected from the total participants, seven significant nutritional principal components (nPCs 1, 2, …, 7; Fig. 1) were extracted. These nPCs 1, 2, …, 7 represented the 90% variation of the total variation in dietary nutrition. nPC1 was extracted as the axis indicating the total amount of overall nutrition (except for the n-6-to-n-3 fatty acid ratio), and nPC2 was extracted as the type of fatty acid (FA), specifically the vegetable oil (positive direction) to animal oil (negative direction) ratio. nPC3 was extracted as the axis representing the total amount of carotene (positive direction) and energy, lipid, and FA (negative direction). nPC4 was extracted as the axis representing the saturated FA ratio (positive direction) and a larger fish oil ratio, greater intake of protein relative to the total energy, and larger vitamin D and mono- and polyunsaturated FA ratio (negative direction). nPC5 was extracted as the axis representing the n-6/n-3 FA ratio (positive direction) and fish oil ratio, vitamin D, and vitamin B12 (negative direction).

#### Pre-process of facial shapes

**Figure 2:**
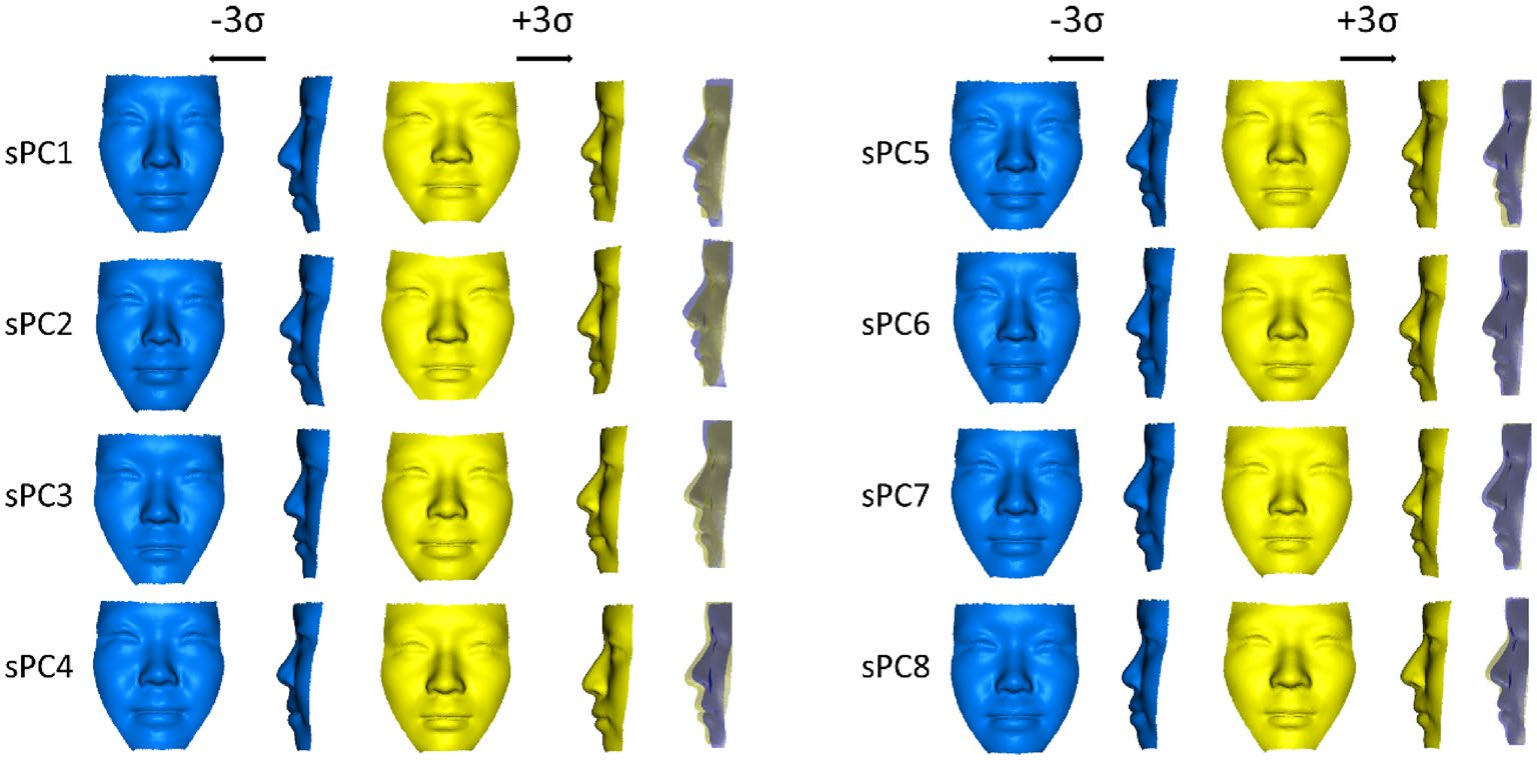
Shape principal components (sPCs) 1–8. Yellow indicates the + 3 standard deviation (σ); blue, − 3 σ. sPCs 1–8 accounted for 70% of the total variation of the faces in the samples. A custom-made MATLAB based software (MATLAB 2021a, The MathWorks, Inc., Natick, MA) was used to create this figure.

The facial shape, with the size of the face eliminated from the original faces, was decomposed into eight significant facial shape principal components (sPCs 1, 2, …, 8, Fig. 2). These were found to explain 70.9% of total samples. A positive value of sPC1 (19%) indicates a greater facial width, midfacial retrusion, protrusion of the chin, and anterior divergent of the face; sPC2 (17%) indicates a concave profile with a greater width of the lower face; sPC3 (10%) indicates a protruded mid-face, greater width of the chin, and flattened forehead; sPC4 (9%) indicates a protruded mid-face, including the base of the nose at a higher position on the nasal base relative to the eyes; sPC5 (8%) indicates a great mandible height and protruded chin; sPC6 (7%) indicates an anteriorly and laterally protruded nose and cheek; and sPC7 (5%) indicates thin lips, a retruded nose, and reduced nasal width.

#### Results of a regression analysis to estimate the nutritional status (dependent variables) based on the facial shape (independent variables) with a multivariate analysis of covariance (MANCOVA)

Three dietary behavior variables (nPCs 4 and 6 and the score representing unhealthy eating behaviors) and four body composition variables (body weight, total muscle mass, total fat mass, BMI) were found to be related to facial shape variables (sPCs 1, 4, 5 and 7) on a stepwise regression analysis (p < 0.05). After these variables were entered into a MANCOVA for adjusted multiple testing, nPC6, body weight, total muscle mass, fat mass, and the BMI were ultimately found to be significantly related to facial shape variables sPCs 1, 4, 5 and 7 (F [7, 98]  = 3.78, p = 0.01, Wilks’ *λ* = *0.79, partial* η^2^ = 0.21, Supplementary Tables S1 and S2).

**Figure 3:**
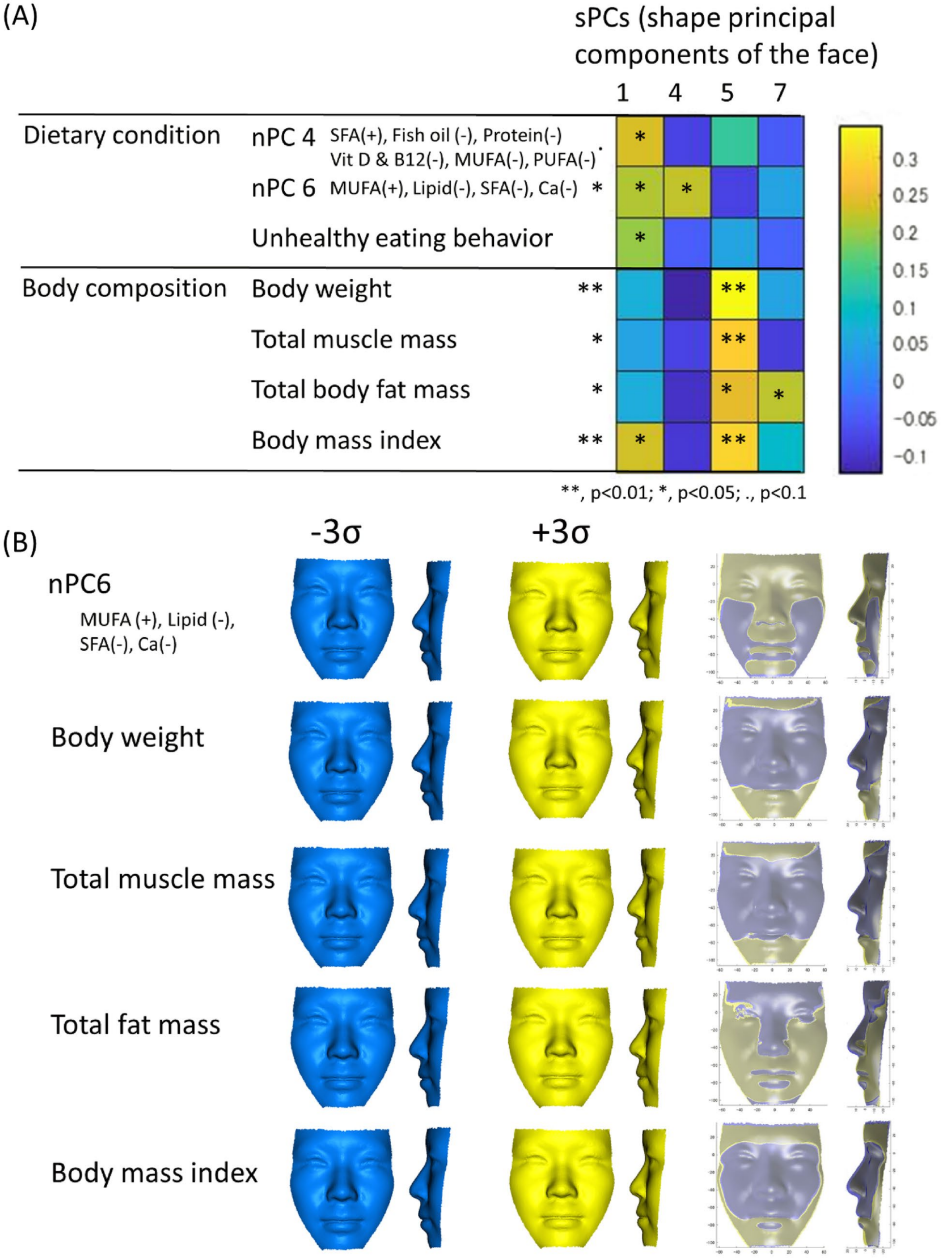
Results of a multivariate analysis of covariance (MANCOVA). (**A**) Heat map of regression coefficients among nutritional principal components (nPCs), dietary conditions, and facial shape principal components (sPCs) and between physical body conditions and sPCs. Blue denotes smaller coefficients and yellow larger values. *SFA* saturated fatty acids, *Vit* Vitamin, *Ca* Calcium, *MUFA* mono-unsaturated fatty acids, *PUFA* poly-unsaturated fatty acids. (**B**) 3D face visualization of nPC6, and body compositions. Blue indicates the − 3 standard deviation (σ); yellow, + 3 σ, which corresponds to 36.4 and 64.6 kg for the body weight, 14.1 and 26.5 kg for the muscle mass, 4.8 and 21.7 kg for the fat mass, and 15.4 and 25.2 kg/m^2^ for the body mass index. A custom-made MATLAB based software (MATLAB 2021a, The MathWorks, Inc., Natick, MA) was used to create this figure.

Regression coefficients of MANCOVA are shown in Fig. 3 as a heat map. The details of the regression analysis are shown in the Supplementary Tables S1 and S2, and Fig. 3 shows the facial visualization of these three dietary condition variables and four body composition variables.

In detail, nPC6 explained 10% of facial variation and was found to be significantly related to sPCs 1 and 4, suggesting that subjects showing a greater consumption of lipids (%energy) with a larger saturated FA ratio and smaller monounsaturated FA ratio (negative direction of nPC6) tended to show a long face due to a higher position of the eyes relative to the nasal base and posterior divergent faces or high angles (negative directions of sPCs 1 and 4, p < 0.05).

The body weight, total muscle mass, body fat mass, and BMI explained 14%, 10%, 12%, and 16% of facial shape variation, respectively. These body composition variables were significantly related to sPC5, thus suggesting that subjects with a large weight, muscle/fat mass, or BMI tended to show a greater mandible height and protruding chin (sPC5, p < 0.05). A greater fat mass was significantly related to thin lips, a retruded nose, and a smaller nasal width (sPC7, p < 0.05). In addition, a large BMI was found to be related to sPC1, suggesting that subjects with a large BMI tended to show an increased facial width, midfacial retrusion, and chin protrusion with anterior divergent faces (sPC1, p < 0.05).

#### Canonical correlations between the nutritional status and facial shape variables

A canonical variate (CV) analysis examining the facial characteristics and nutritional contents showing maximum correlation between them found significant canonical correlations between the nutritional status and face (first canonical variate [CV 1], F [76,341.15] = 1.48, p (F) = 0.01, Wilks’ *λ* = *0.32, r* =  + *0.60*).

**Figure 4:**
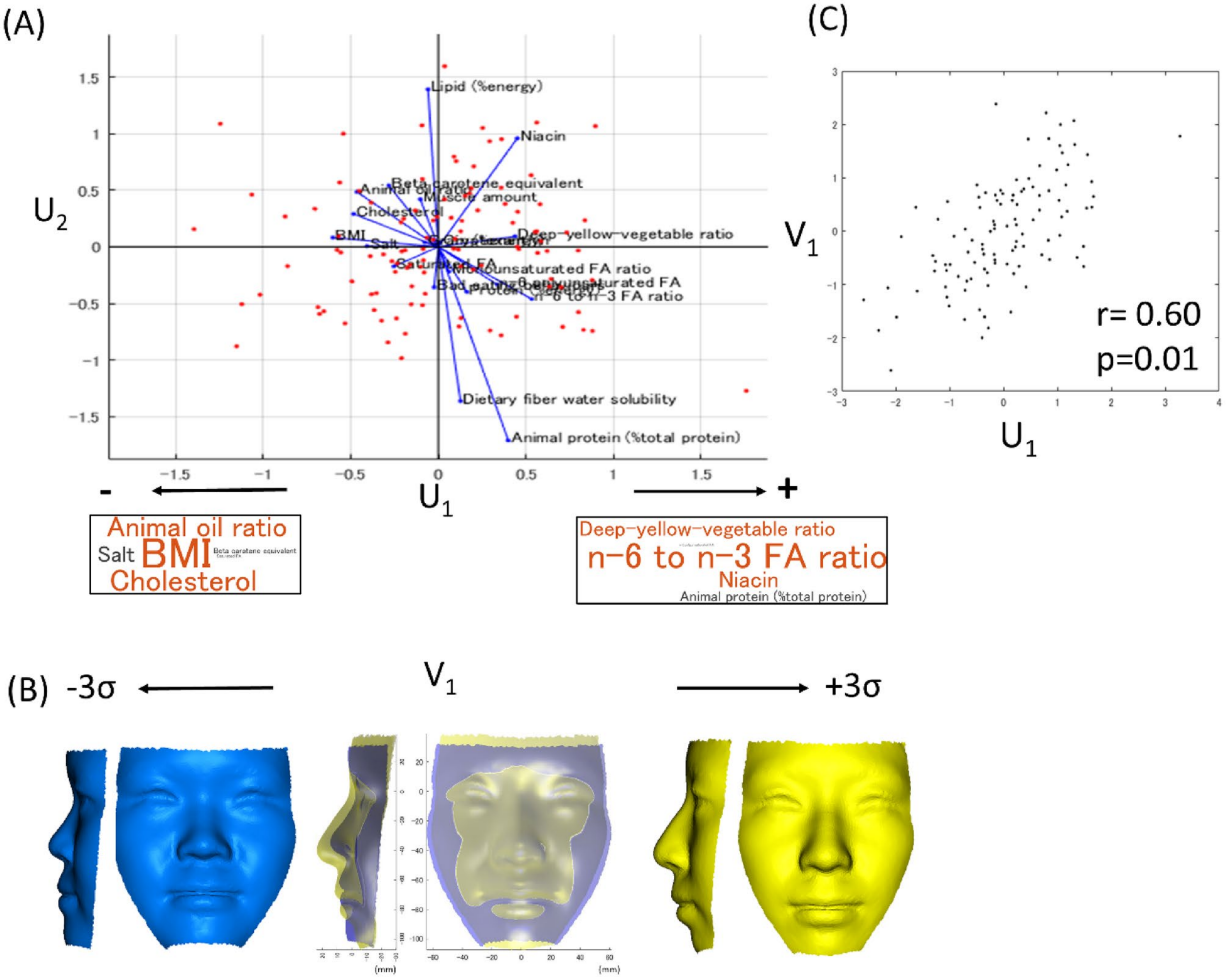
Result of a canonical variate (CV) analysis. A significant correlation was found in the first CV (CV 1, p < 0.01, r = 0.60). (**A**) Nutritional axes of CVs 1 and 2 (U_1_ and U_2,_ respectively). U_1_ was associated with a greater intake of n-6 polyunsaturated FA relative to n-3 unsaturated FA, deep-yellow-vegetable relative to the total vegetables, and niacin. In contrast, the negative direction of the U-axis was associated with a larger BMI and greater intake of dietary cholesterol, animal oil relative to total oil, and salt. (**B**) Visualization of the facial axis of CV1 (V_1_). A CV analysis showed that V_1_ was explained by shape principal components (sPCs) 1, 4, 5, and 7 with standard coefficients of − 0.67, + 0.34, − 0.56, and − 0.34, respectively. This means that the V-axis was associated with a smaller facial width, midfacial protrusion, posterior divergent face (sPCs 1, 4, and 7), lower position of the eyes (sPC4), smaller mandible height and retruded chin (sPC5), thick lips, and greater nasal width (sPC7). (C) A scatter plot of U_1_ (nutrition) and V_1_ (facial shape). A custom-made MATLAB based software (MATLAB 2021a, The MathWorks, Inc., Natick, MA) was used to create this figure.

Visualization of the correlated nutritional axis and faces is shown in Fig. 4. In short, the nutritional status explained by a greater BMI, greater intake of dietary cholesterol, animal oil relative to total oil, and salt was significantly correlated with the facial characteristics of a greater facial width, midfacial retrusion, protrusion of the chin, an anterior divergent face (sPCs 1, 4, and 7), a lower position of the nasal base relative to the eyes (sPC4), smaller mandible height (sPC5), thick lips, and a greater nasal width (sPC7). These results were mostly consistent with those of the MANCOVA.

### Indirect comparisons between nutritional status and 3D facial shapes

#### Clustering of the subject groups based on holistic dietary conditions

To quantify the 3D facial forms when maximizing the differences between distinct dietary intake patterns, the mathematical clustering method was used to subclassify the participants into three subject groups (Codes) based on the nutritional intake and eating behavior scores. The results are summarized in Table 1 and shown in Supplementary Tables S3–S8 as well as Supplementary Texts S1–S3. In short, Code 1 (n = 42), Code 2 (n = 41), and Code 3 (n = 32) were labeled as a “balanced low-calorie dietary pattern”, “high-calorie dietary pattern”, and “imbalanced low-calorie dietary pattern,” respectively, based on the significant differences in multiple comparisons among Codes, as shown in Table 1 and described below.

**Table 1:** Summary of the dietary intake and eating behavior and the corresponding physical condition, including facial characteristics.

Variables	Code 1 (n = 45^41^)	Code 2 (n = 36^35^)	Code 3 (n = 34^33^)
	Balanced low-calorie-diet pattern	High-calorie-diet pattern	Imbalanced low-calorie-diet pattern
**Nutritional contents**	Fish oil ratio (+) Carbohydrate (−) Salt/Sodium/Phosphorus (−) Retinol (−)	Energy (+) Carbohydrate (+) Protein (+) Lipid (+) Saturated/monounsaturated fatty acid (FA) (+) Fish oil ratio (+) Cholesterol (+)	Carbohydrate (+) Animal protein ratio/protein (−) Fish oil ratio (−) Vegetable oil ratio (+) n-3 polyunsaturated FA (−) n-6/n-3 FA ratio (+) Vitamin D/B12, Niacin (−)
		Salt/Sodium/phosphorus (+)	Monosaturated FA ratio (−)
		Vitamin B1/ B2/ B6 (+) Niacin (+) Pantothenic acid (+) Tocopherol (+)	
		Mineral, calcium (+)	
**Nutritional principal components (nPCs)**	nPC4(−) [greater fish oil ratio, greater intake of protein relative to the total energy]	nPC1 (+), nPC3 (−) [greater total amount of overall nutrition (nPC1), greater intake of energy, lipid, and FA total amount (nPC3)]	nPC2 (+), nPC5 (+) nPC6(−) [greater vegetable oil ratio (nPC2), greater n-6/n-3 FA ratio (nPC5), greater consumption of calcium, lipid (%energy), and saturated FA ratio (nPC6)]
**Eating behavior**			
Bad recognition of weight	(−)	(+)	(+)
Bad external eating behavior	(−)	(+)	
Bad emotional eating behavior	(−)	(+)	(+)
Bad sense of hunger	(−)	(+)	
Bad eating style	(−)	(+)	(+)
Bad food preference	(−)	(+)	
Bad regularity of eating habits	(−)	(+)	
**Body compositions**			
Body mass index (BMI)	(−)	(+)	(+)
Fat-free mass index (FFMI)^33^	(−)	(+)	(−)
Fat mass index (FMI)^33^, body fat (%)	(−)	(+)	(+)
Body weight, muscle mass	NS	NS	NS
**Face**	Smaller zygomatic width (|Zy-Zy|)	Lower position of the Nasion when compared with the eyes (|N-En|)	Greater lower face height and mandibular height (|Sto-Gn|, |Gla-Sn|/|Sn-Gn|)
	Smaller gonial width (|Go-Go|)	Laterally greater facial width relative to the height (|N-Gn|/|Zy-Zy|, |Gla-ls|/|Zy-Zy|)	Smaller eye height (|Ps-Pi|)
**Shape principal components**			
sPC1 (increased width and reduced height of the face)	(−)	(+)	NS

For details, please see Supplementary Tables S1–S3.

(−) denotes a significantly smaller value than the other 2 groups; (+) denotes a significantly greater value than the other 2 groups; NS, not significant (ANOVA, p < 0.05, post-hoc test, Tukey–Kramer). [] indicates the number of faces used.

Both Codes 1 and 3 were low-calorie-diet patterns, but Code 3 was characterized by less animal-related nutrition (e.g. reduced intakes of n-3 polyunsaturated FAs, vitamin D, vitamin B12, niacin, and animal protein), more vegetable-related nutrition (e.g. greater vegetable oil ratio, carbohydrate, n-6 polyunsaturated FAs^32^), a preference for sugar and fatty foods, emotional eating behavior, a faster eating style, night eating, and the recognition of easy weight gain.

Regarding body composition, both Codes 2 and 3 showed a greater BMI and fat mass than Code 1 (p < 0.05), while the body weight and muscle mass did not show significant differences among the three Codes. Code 3 showed the highest fat percentage among the groups (p < 0.01).

Furthermore, the additionally calculated fat-free mass index (FFMI, fat-free mass/height^2^) and fat mass index (FMI; fat mass/height^2^)^33^ indicated that a high-calorie-diet showed a greater FFMI than Codes 1 and 3, and Code 3 showed a greater FMI than Code 1, indicating that Code 3 is classified as a “sarcopenic obesity tendency” and Code 2 is classified as an “obesity tendency”^33^. We use the word “tendency” here to describe the slight differences in normal-weight subjects, as 99% of subjects were considered either underweight or normal weight.

There were no significant differences among the code groups in physical activity level, duration of physical exercises, birth weight, 2-year change in weight, sleep duration, or menarche age for the 43 randomly selected participants (p > 0.05, Supplementary Text S4, Supplementary Table S9).

#### 3D soft-tissue facial forms specific to each pattern

**Figure 5:**
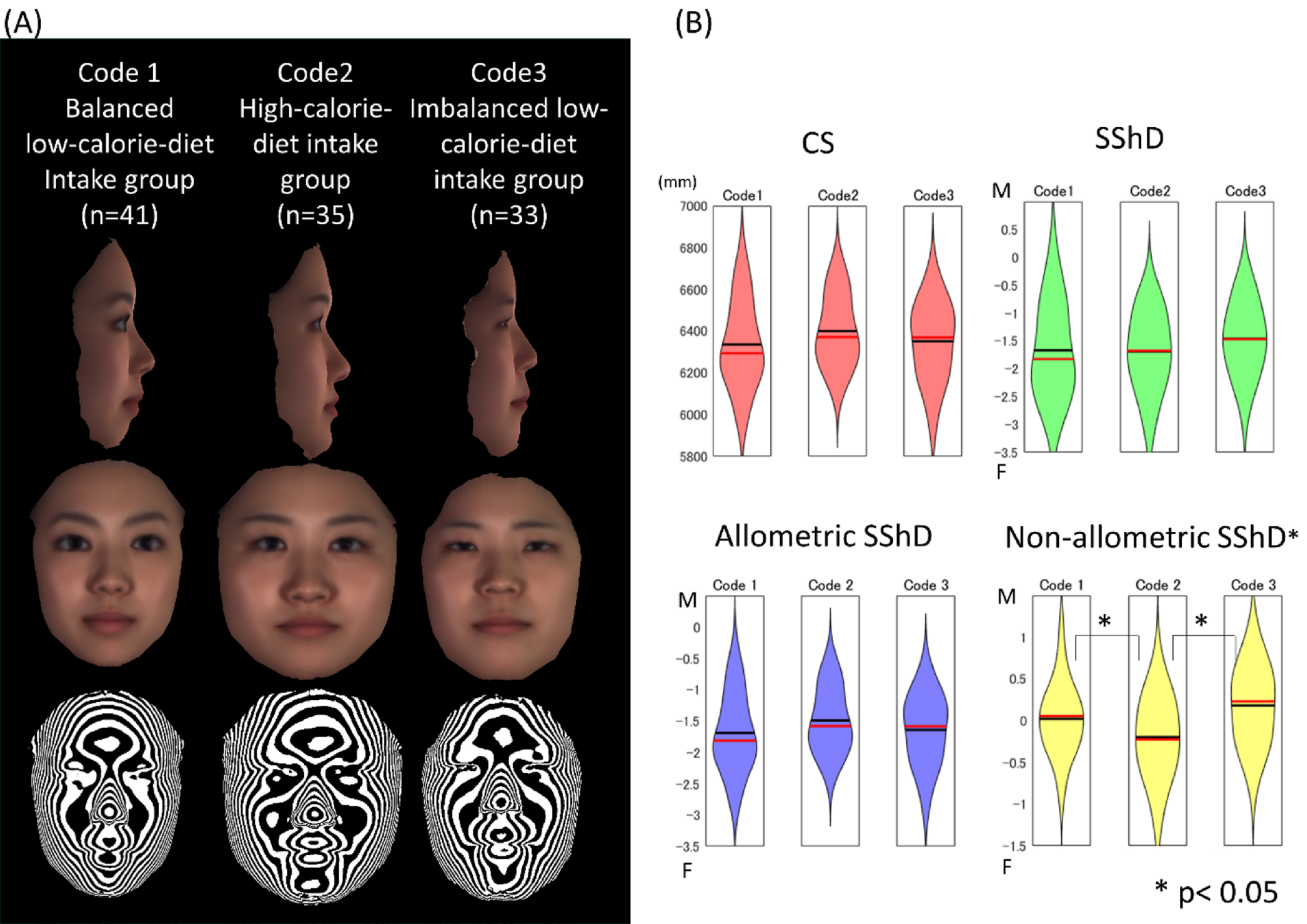
(**A**) The accentuated averaged faces for the three code groups determined in the present study; and (**B**) centroid size (CS), sexual shape dimorphism (SShD), allometric SShD, and non-allometric SShD. (**A**) For the computational procedure, see Tanikawa et al.^30^, where five was used as a weight to accentuate the differences among the codes. Top, lateral view; middle, frontal view; bottom, Moire image. Three facial images in Code 1, three facial images in Code 2, and two facial images in Code 3 showed insufficient data quality in the region of the ears and were thus excluded from the analysis. This insufficient data quality was due to some participants not perfectly removing their hair from around their ears, which was necessary for taking good-quality images with the 3D cameras. (**B**) M indicates hyper-masculine face, and F, hyper-feminine face. *p < 0.05 (ANOVA, Turkey-Kramer post-hoc test). A custom-made MATLAB based software (MATLAB 2021a, The MathWorks, Inc., Natick, MA) was used to create this figure.

The facial characteristics specific to each pattern were visualized in Fig. 5.

The non-allometric SShD of Code 3 was significantly greater than that of Code 2, and that of Code 2 was significantly smaller than that of Code 1 (p = 0.004, F = 5.83, η^2^ = 0.1), indicating that an imbalanced-diet pattern was associated with a ‘masculine face’, whereas a high-calorie-diet pattern was associated with a ‘feminine face’. There were no significant differences in the CS, SShD, or allometric SShD among the Codes.

A detailed landmark-based analysis (Supplementary Table S7)^34^ clarified that a Code 1 balanced diet pattern showed smaller zygomatic and gonial widths than the other two patterns.

The Code 2 high-calorie-diet group showed a greater zygomatic width to facial height than Code 1. In addition, Code 2 also showed a lower vertical position of the nasal bridge relative to the eyes than Code 1 and Code 3 subjects, indicating a low and depressed nasal bridge^35^. A low nasal bridge is generally related to a short nose, flat nasal bridge, and flattened midface^36^. These results suggest that a high-calorie-diet pattern led to a greater facial width with midfacial retrusion and protrusion of the chin than the other two groups, results that were almost concordant with those of the MANCOVA (body compositions) and negative direction of the first CV of the CV analysis.

The Code 3 imbalanced low-calorie-diet group showed a significantly greater mandibular height than the other two groups. Code 3 was also characterized by a smaller eye height than the other two groups.

Relationships between the nutritional status and facial characteristics observed in Code 3 corresponded to the findings concerning a positive direction of nPC6 on the MANCOVA (greater consumption of lipid [%energy] and calcium with a smaller intake of monounsaturated FA ratio) and a positive direction of the first CV of the CV analysis.

## Discussion

In the present study, we demonstrated that the nutritional status was associated with facial morphology in young Japanese women in three multivariate analyses (MANCOVA following PCA, CV analysis, clustering analysis). In particular, the clustering analysis determined three major dietary patterns in this population: the balanced low-calorie-diet pattern (Code 1), high-calorie-diet pattern (Code 2), and imbalanced low-calorie-diet pattern (Code 3). All of these analyses consistently showed two results: (1) high-calorie-diet pattern showed greater facial width, midfacial retrusion, protrusion of the chin, and anterior divergent face than a balanced diet pattern. In contrast, an imbalanced-diet pattern, which was mainly characterized by a preference for sugar (carbohydrate) and fatty foods but low energy, showed an increased lower facial height and small eye height. The imbalanced-diet pattern was confirmed to be related to biological ‘masculine’ facial characteristics using geometrics morphometrics. Body composition showed that the imbalanced-diet pattern was characterized by a sarcopenic obesity tendency, and the high-calorie-diet pattern was characterized by a simple obesity tendency. The balanced-diet pattern was characterized by a normal body composition. The details are described below.

Thus far, several studies have shown that Western dietary intake patterns are significantly associated with obesity, insulin resistance (a status of inability of insulin to increase glucose uptake and utilization^37^), and metabolic syndrome, which are risk factors for cardiovascular disease^38^. A Western dietary intake pattern is characterized by a high intake of red meat, processed foods, sweets, dairy and refined grains, and soft drinks with a low intake of other vegetables^38^ according to several studies using clustering or factor analyses^39^. In the present study, a Code 2 high-calorie-diet pattern corresponded to a typical Western dietary intake pattern because of the preference for fatty foods, snacks, sweets, and meat. Excess calories can be stored in fat cells, which seems to have resulted in the increased BMI and facial adiposity in the high-calorie-diet pattern in the present study. Visualization of the high-calorie-diet pattern helped show that this group had facial adiposity or a rounded appearance due to fat deposits on the sides of the face (Fig. 5).

These results are in line with several previous studies regarding facial appearance in 2D^10^. A previous study using 2D photos from 22 women showed that the subjects with a high body fat proportion had a larger lower face with a wider jaw outline, shorter and wider nose, fuller lips, and more downturned mouth corners than those with less body fat^11^. Another study that used 2D photos from 49 women showed that the faces of women with a high BMI had a wider and rounder facial outline relative to the size of the eyes and lips and lower eyebrows than those with a low BMI^11^. Another study using a total of 194 2D photos consisting of 2 groups of Caucasian women and two groups of African women showed that the width-to-height ratio was associated with the BMI^40^. A study using 2D photos from 526 women showed that the greater BMI group showed reddish, round faces^15^. In the present study, fuller lips and wider nose were not observed in the high-calorie-diet group, but the other facial characteristics previously observed in increased BMI subjects were consistent in the present study.

It was assumed that a high-calorie-diet pattern was related to not only soft-tissue but also hard-tissue craniofacial shapes. Cephalometric research has shown correlations between facial shape and the BMI. A previous study using 50 adolescents^41^ showed a greater maxillary and mandibular length (by 3–3.5 mm and 8–10 mm, respectively) in the obese group than in controls. A mandibular height was found to be greater in the obesity group than in the controls, and the mandibular plane angle was smaller in the obesity group than in the control group, resulting in less convex profiles^41^. This is consistent with the present results concerning midfacial retrusion and posterior divergence of the face in the high-calorie-diet pattern and with a negative direction of CV1. Because the previous results regarding hard-tissue were obtained in children and adolescents, whether or not these cranio-facial characteristics will hold through adulthood is unclear. However, it is still possible that the current findings of less facial convexity (chin protrusion and a retruded midface) with the high-calorie-diet pattern may be due to the skeletal influence of the nutritional status. Further studies regarding the influence of the nutritional status on the hard tissue will be needed.

Regarding the mechanism underlying the changes in facial shape with a high-calorie-diet pattern, sex hormones might be involved. In the present study, we found a significantly ‘feminine’ facial shape compared with an imbalanced-diet pattern using non-allometric SShD. This is due to the fact that biological non-allometric feminine features are characterized by an antero-posteriorly small nose (midface) and increased sagittal cheek protrusion in the infraorbital region^30^. There have been many studies showing that Western-type dietary patterns are consistently associated with higher estrogen concentrations in premenopausal and post-menopausal women^39,42^. This is because adipose tissue is a major site for metabolism of sex steroids, and adipose tissue contributes up to 100% of estrogen in postmenopausal women and approximately 75% of total estrogens in premenopausal women^43^. Furthermore, a greater dietary energy intake was inversely associated with androstenedione and dehydroepiandrosterone sulfate (DHEAS) in premenopausal women^44^. Thus, higher estrogen and lower androstenedione and DHEAS levels might be related to the facial characteristics in the high-calorie-diet pattern. A study measuring the plasma estrogen level showed that 2D average facial pictures for subjects with high estrogen levels shared similar features to our high-calorie-diet pattern subjects^45^. Thus, the biologically feminine facial shape observed in the present study may be related to elevated estrogen levels. Further studies are needed to confirm the hormone levels in each pattern.

Regarding the present finding that the presence of facial adiposity with an increased BMI was associated with a feminine facial morphology, several psychological studies have shown contradictory results; namely, an increased BMI in women was concluded to be associated with perceived ‘maleness’ of the face^18,46^. This contradiction can be explained by two hypotheses. First, the previous psychological study was based on ‘perceived’ maleness facial characteristics, regardless of the evidence that the shape features perceived as masculine only partly resembled the biological facial sexual dimorphism characteristics^26^. Indeed, consistent with these psychological studies, the present findings also showed a significantly increased facial width-to-height, which has been used as a feature indicating increased perceived ‘maleness’ in psychological studies. Second, the ‘obesity’ group used in previous psychological studies may have included subjects with a high-calorie-diet obesity tendency as well as those with an imbalanced-diet sarcopenic obesity tendency, both of which show an increased BMI. Further psychological studies with isolating these obesity types will help resolve this contradiction.

Regarding the imbalanced low-calorie-diet pattern (Code 3), the pattern was mainly characterized by a preference for sweets and fatty foods and a decreased dietary intake of animal protein (including fish) and Vitamins D and B12. The results indicated that this type of eating behavior can cause a sarcopenic obesity tendency. Sarcopenic obesity is primarily used to describe older individuals, being defined as a low skeletal muscle mass relative to the total body mass^47^. However, sarcopenia has recently been reported to be related to disease susceptibility, even in healthy young adults^48^. Young Japanese women have shown a particularly high prevalence of pre-sarcopenia at 36%^49^. A previous study showed that sarcopenic obesity in young women was often (53%) observed in women with polycystic ovary syndrome (PCOS)^50^, where PCOS is a very common endocrine disorder in young women, with an estimated prevalence of 5% to 12% in premenopausal women. Clinical features associated with this syndrome include hyperandrogenemia, reproductive dysfunction, insulin resistance, and hyperinsulinism^51^. Furthermore, previous studies^50,52^ have shown that Vitamin D deficiency is related to metabolic risk factors in POCS patients. Thus, while only an assumption, we may reasonably suspect that a sarcopenic obesity tendency in combination with Vitamin D deficiency due to an imbalanced low-calorie-diet pattern is associated with a high biological level of androgen, causing ‘masculine’ faces.

Although the mechanisms for sarcopenic obesity and a high androgen profile remain somewhat unclear, increased liver fat^53^ and decreased levels of sex hormone-binding globulin (SHBG)^54^ may be involved. A qualitative decline in muscle mass and an increase in fat mass with sarcopenic obesity may cause an increase in the amount of liver fat (nonalcoholic fatty liver)^55^. Further, this increased amount of liver fat is likely to cause a decrease in SHBG, which is produced and secreted by the liver into the bloodstream^53^. Because SHBG binds to sex steroids and thereby regulates their bioavailability^56^, decreased SHBG levels are related to an increased bioavailability of androgens^57^. The association between nonalcoholic fatty liver disease and sarcopenia was found to be stronger in younger groups than in older ones^58^. Thus, it is reasonable to assume that the sarcopenic obesity tendency of the present imbalanced-diet pattern in young women may cause an increased bioavailability of androgen.

Finally, the balanced low-calorie-diet pattern (Code 1) was characterized by a low energy intake with the lowest intake of carbohydrate (e.g. sugar) among all three groups, well-balanced intakes of macronutrients, and the greatest intake of fish oil (n-3 polyunsaturated FAs). These characteristics suggest that the balanced-diet pattern had a diet including fish (i.e. the traditional Japanese diet). The Japanese consume on average more than 100 g of fish every day from childhood^59^. The current understanding regarding the possible effects of dietary n-3 polyunsaturated FAs on adiposity in humans is limited, although high intakes reportedly reduce the degree of fat accumulation^60^. It has also been documented that rats fed a high-fat diet supplemented with fish oil had less fat in their perirenal and epidydimal fat pads and decreased adipocyte volumes than those fed lard. It appears reasonable to assume that a reasonable intake of n-3 polyunsaturated FAs may contribute to obesity prevention. n-3 polyunsaturated FAs also exert beneficial effects on the skeleton, as n-3 supplementation is associated with bone mineral density at various locations in mice^61^. They also help to prevent bone loss induced by ovarian resection^62,63^. These observations may primarily be due to the specific inhibitory effects of docosahexaenoic acid on osteoclast differentiation, activity, and the systemic inflammatory environment^64^. Randomized controlled trials have reported the association of muscle mass and performance with specific nutrients, such as n-3 FAs^65^. A previous study^66^ showed that n-3 polyunsaturated FAs reduced the levels of plasma inflammatory cytokines. The slim facial proportion, brighter eyes, shapely nose, and elevated angles of the mouth in the balanced-diet subject group observed in Fig. 5 may well explain the possible effects of increased n-3 FAs combined with healthy eating behaviors on facial shape.

In the present study, we examined eating behaviors using questionnaires. Leptin, an adipocyte-derived hormone, acts on neurons in certain brain areas and strongly inhibits feeding behavior, and obese subjects sometimes demonstrate leptin resistance. Leptin resistance is defined by a reduced sensitivity or failure in response of the brain to leptin, showing a decrease in the ability of leptin to suppress appetite or enhance energy expenditure, thus causing increased food intake and finally leading to overweight and obesity. As a result, the increased scores in appetite-related eating behaviors shown in the present study may suggest increased leptin resistance. As leptin is an adipocytokine secreted by adipose tissue, this explanation concerning an increased appetite in both unhealthy groups appears reasonable.

The present study employed data-driven methods. The method used in the present study resembles the recently developed model used in the Environmental-Wide Association Study (EWAS). The EWAS was proposed to identify multiple environmental factors (173 to 266 nutritional and lifestyle factors) associated with complex diseases (e.g. deficiency testosterone^67^, peripheral arterial disease^68^) by borrowing the GWAS methodology, including a data-driven analysis. To our knowledge, the present study was the first to examine the 3D facial morphology based on multiple environmental factors (i.e. dietary intake, eating behavior, and body compositions) using a data-driven method. As described previously^69^, it is difficult to explain the facial environmental factors using only the present data-driven research. Further classical hypothesis-driven research would be beneficial to confirm the present results.

Several limitations associated with the present study warrant mention. First, the sample size included in our analysis was relatively small; it is possible that our results would have differed with a larger sample. Second, our sample population had limited variation in age (mean age, 20 years old) and ethnicity (Japanese). Therefore, readers should consider these sample variations when applying our findings to different populations. Third, our study included mainly underweight and normal-weight subjects. A wide range of samples should be considered in future studies. Fourth, previous studies also proved that the dietary intake influenced the skin appearance, specifically the skin color^70^ and skin-aging appearance^71^. Further research, including that incorporating facial skin color, would help clarify the influence of eating behaviors on facial appearance. Furthermore, our Japanese samples were mainly (81%) from the local Kinki area. A previous study showed that the populational origin of Japanese subjects was related to specific facial morphological characteristics (i.e. the indigenous hunter-gathering population Jomon ancient has a short face, while the rice-farming population Yayoi ancient has a long face^72^), but we did not consider the hometown as a confounding factor. This is because gene examinations in Japan have estimated that the Kinki area population has a Yayoi contribution of 80–90%, and other Japanese areas with high Jomon contributions (North and South areas of Japan) also show relatively high Yayoi contributions of 70–80%^73,74^, and it was difficult to specify the genetic origin population of each participant. Our results showing that the hometown (whether participants lived with their families or not) was not related to the eating behavior pattern (Supplementary Text S4) indirectly indicate that the hometown can be excluded as a confounding factor when examining relationships between dietary behavior and facial morphology. However, future research should confirm whether or not the specific populational origin is related to facial characteristics and eating behaviors. Finally, the present study did not measure sex hormones directly, and the possible existence of hormonal influences was only discussed based on the SShD (sex differences of the face). Further research that measures sex hormones will be needed to directly clarify the link between sex hormones and facial morphology.

Once eating behaviors are learned incorrectly, their nutritional effects on an individual’s health accumulate over a lifetime. Thus, learning proper eating behaviors based on scientific evidence is important, especially in developed countries, where individuals can make their own choices about foods and eating styles. The recognition of facial changes associated with eating behaviors achieved in the present study may be effective in motivating individuals to correct their eating behaviors. Furthermore, the present study suggests the potential utility of 3D facial images for estimating the body’s condition due to nutritional influences. Since 3D images of the face include a large amount of information and can be captured even with smartphones these days, they may be useful in our daily lives for maintaining one’s health status or changing one’s eating behaviors in the future, as research on the use of artificial intelligence to estimate the nutritional status and body composition progresses.

In summary, the amount and patterns of macro- and micro-nutrient intake, eating behaviors, body composition were related to the 3D facial morphologies of young Japanese women. The present study discussed the possibility that biological aspects (e.g. obesity, sex hormones) that can be altered by the nutritional status may influence the facial morphology.

## Methods

The present study was approved by the Ethics Committees at Mukogawa Women’s University (No. 12-30). All experiments were performed in accordance with relevant guidelines and regulations.

### Subjects

The participants were 115 female university students with an average age of 20.6 ± 0.48 (range 19.3–23.2) years old. We recruited participants from School of Human Environmental Sciences, Mukogawa Women's University (Nishinomiya, Japan) which is located in an urban area, to eliminate variations in educational status and residential area. The data were collected from May 2013 and May 2014. A written informed consent form was distributed to and signed by all participants. Informed consent was approved by the Ethics Committees at Mukogawa Women’s University (No. 12-30). No relatives were included among the subjects.

### Data recording

#### 3D facial shapes

For the morphological characteristics of the soft tissue faces, 3D facial images were noninvasively recorded for each participant, using a 3D image-capturing device (3dMDcranial Systems; 3dMD LLC, Atlanta, GA, USA). Each participant sat upright in a chair with no back support. While recording, each participant was asked to keep the teeth in light intercuspation and the lips in repose.

#### Holistic dietary conditions

In the present study, we examined the participants’ holistic dietary conditions by combining information from the nutrition contents in daily food intake and eating behaviors.

#### Nutrition contents in daily food intake

Daily food intake was recorded for each participant using a food frequency questionnaire on food groups^75^, which comprised items from 29 food groups and 10 types of cooking, and calculated the average intake per week of each food or food groups in commonly used units or portion sizes. In detail, the participants were instructed to fill out questionnaires in which respondents described their consumption of 29 food groups and 10 types of cooking with regard to the amount (“A little”, “Normal”, and “Plenty”) and frequency (how many times a week). Regarding the amount of foods, visual aids (illustration and food models) were used to avoid bias concerning the actual portions. To estimate the intake of sugar, salt, and oils, questions about cooking methods (e.g. boiled, deep-fried, fried, and soup) were also collected. The survey time was 30 min for each participant. After the participants completed answering the questionnaire, three nationally registered dieticians (MK, NT, and MT) interviewed each participant to review the completed questionnaire. The nutrient intake was then estimated using an Excel software program add-in (Excel Eikun Ver. 6.0; Kenpakusha, Tokyo, Japan) based on the Revised Standard Tables of Food Composition in Japan^76^ as follows:

Nutrient intake = Portion size per food group (gram [g]) × Food amount category value × Number of times consumed in a week/7 days × Amount of each nutrient per gram of food group in the average composition table^76^.

In this formula, the food amount category value was defined as follows: "No" = 0, "A little" = 0.5, "Normal" = 1, "Plenty" = 1.5. To estimate salt consumption, a question comparing the taste of each household's condiments to that found in restaurants was also included, and the salt amount was adjusted by multiplying by 1.15 (equal to restaurant food) and 1.3 (saltier than restaurant food). Validation of this estimation approach was confirmed in a previous study^75^.

Estimated nutrients included the following 53 nutrient items: energy [E] (kcal), grain (%E), water (g), protein (g), protein (%E), animal protein ratio (% total protein), lipid (g), lipid (%E), FA total amount (g), saturated FA (g), cholesterol (mg), monounsaturated FA (g), polyunsaturated FA (g), n-3 polyunsaturated FA (g), n-6 polyunsaturated FA (g), carbohydrate (g), carbohydrate (%E), deep-yellow-vegetable ratio, dietary fiber water solubility (g), dietary fiber insolubility (g), dietary fiber total amount (g), retinol (μg), alpha carotene (μg), beta carotene (μg), cryptoxanthin (μg), beta carotene equivalent (μg), retinol equivalent (μg), vitamin D (μg), alpha tocopherol (mg), beta tocopherol (mg), gamma tocopherol (mg), delta tocopherol (mg), tocopherol equivalent (mg), vitamin K (μg), vitamin B1 (mg), vitamin B2 (mg), Niacin (mg), vitamin B6 (mg), vitamin B12 (μg), folic acid (μg), pantothenic acid (mg), vitamin C (mg), mineral (g), sodium (mg), salt (g), potassium (mg), calcium (mg), magnesium (mg), phosphorus (mg), iron (mg), zinc (mg), copper (mg), and manganese (mg). In addition, six ratios (animal oil ratio, vegetable oil ratio, fish oil ratio, saturated FA ratio, monounsaturated FA ratio, polyunsaturated FA ratio, and n-6/n-3 FA ratio) were calculated based on the Revised Standard Tables of Food Composition in Japan^76^.

#### Eating behaviors

Eating behaviors or dietary habit was also recorded using the questionnaire, based on the guidelines for obesity issued by the Japan Society for the Study of Obesity^77^. The questionnaire consisted of 55 questions consisting of 7 major categories (i.e. recognition of weight and constitution, external eating behavior, emotional eating behavior, sense of hunger, eating style, food preference, regularity of eating habits). Questions are shown in Supplementary Table S5. All items were rated on a four-point scale ranging from 1 (i.e. “seldom”) to 4 (i.e. “very often”).

### Physical body composition

Four body composition parameters (body weight, total skeletal muscle mass, total body fat mass, and BMI) were measured using a multifrequency bioelectrical impedance analysis device (InBody 720; Biospace, Inc., Tokyo, Japan)^78^. Further, body fat percentage (fat mass/weight), fat-free mass index (FFMI, fat-free mass/height^2^) and fat mass index (FMI; fat mass/height^2^)^33^ was determined from measured body compositions.

The samples’ mean weight and BMI were 50.97 ± 4.92 (range 37.64–63.38) kg and 20.29 ± 1.67 (range 16.94–25.40) kg/m^2^, respectively. The reported average weight and BMI in Japanese women (18–29 years old) is 50.0 kg and 20.0 lg/m^2^, respectively^76^, indicating that the mean of the sampled populations was almost equal to the national values. Based on the BMI classification of ‘normal weight’ as 18.5–24.9 kg/m^2^^79^, the proportions of participants categorized as underweight, normal weight, and pre-obesity were 15%, 86%, and 1%, respectively. These data indicate that the sample was slightly thinner but otherwise representative of young Japanese women.

Relationships between body compositions and nutritional intakes or eating behaviors were shown in Supplementary Text S5.

### Analyses

Data were analyzed via two methods: (1) direct comparisons between 3D facial shapes and eating behaviors and (2) indirect comparisons between these items. (1) was achieved by a multivariate analysis of covariance (MANCOVA) and canonical variate (CV) analysis, while (2) was conducted by clustering participants based on their eating behaviors and visualizing the averaged 3D faces of each cluster. All analyses were conducted using the statistical software program included with R (http://www.r-project.org/) and MATLAB (MathWorks, Ltd., Natick, MA, USA).

#### Direct comparisons between 3D facial shapes and the dietary condition

We directly compared the 3D facial shapes and dietary conditions (i.e. nutritional conditions and eating behavior) in three steps: a geometric morphometrics analysis of 3D faces, a summary of the nutritional conditions, and an examination of the relationships between facial shapes and eating behaviors. The details are described below.

### Geometric morphometrics analyses of 3D faces

A 3D coordinate system identical to that employed in our previous study (Supplementary Fig. S1; Informed consent was obtained to publish this image in an online open-access publication.)^34^ was used in the current study. In short, the sagittal plane was defined by exocanthions and endocanthions, and the axial plane was defined by exocanthions, the porion, and the subnasale. The nasion was set as the origin.

For each facial surface, fitting of high-resolution template meshes^34,80^ was performed using a commercial software program (HBM-Rugle; Medic Engineering Co., Kyoto, Japan) based on the landmarks assigned to each 3D image (Supplementary Table S10). This method automatically generated a homogeneous model that consisted of 6017 Cartesian semi-landmarks on the wire mesh for each model. This technique permits the extraction of relevant surface anatomy from face data while removing non-relevant data, yielding 3D surface data that provide enough detail to facilitate a quantitative assessment while minimizing file sizes to still be sufficient to represent the facial shape (Supplementary Fig. S2; Informed consent was obtained to publish this image in an online open-access publication). The 6017 Cartesian semi-landmark coordinates were analyzed by geometric morphometrics. In brief, after separating the shape from the overall size, position, and orientation of the landmark configurations, the resulting Procrustes shape coordinates were used for subsequent statistical analyses, where the overall size was calculated as the centroid size (CS). To examine the variance in facial shapes of the subjects, we performed a principal component analysis (PCA) for the 6017 coordinates of the aforementioned surface model. The dimensionality of the shape principal components (sPCs) was determined to include sPCs with eigenvalues greater than 1, namely the Kaiser criterion. The facial morphospace (new dimensional space representing facial shape) consisted of these significant sPCs and used in the following process:

### Summarization of the nutritional conditions

To examine the variance in nutritional conditions, the estimated nutrient items included in the daily food intake were dimensionally reduced using the PCA. The dimensionality of the nutritional principal components (nPCs) was also determined using the Kaiser criterion, as above. For both sPCs and nPCs, if the cumulative contribution rate based on the Kaiser criteria was greater than 90% variations our of all the variations, then we used the 90% cumulative contribution rate of total variance as the cutoff to determine the number of PCs.

### Relationships between facial shapes and dietary conditions

To examine the direct relationship between facial shapes and dietary conditions, sPCs that corresponded to nPCs, eating behavior variables, and physical conditions (body weight, total skeletal muscle mass, total body fat mass, and BMI) were examined with a stepwise regression analysis, as follows:

$$V = w_{1} \times {\text{sPC1}} + w_{2} \times {\text{sPC2}} + \cdots + w_{k} \times {\text{sPCk}}$$

where k indicates the number of the sPCs; *w*_*1,2,…k*_ indicates the coefficient values of the regression analysis, and *V* is each nPC, eating behavior score, and physical condition (dependent variables). The inclusion criteria for the stepwise analysis were set as items with p-values of 0.05. To control multiple testing, significant dependent variables and selected sPCs were entered into the MANCOVA. The regression coefficient was represented using a heat map.

Further, a CV analysis was conducted to examine if correlation is significant between the facial characteristics (sPCs) and the dietary intake amount of nutritional contents when showing maximum correlation between them. Faces showing maximum correlations were visualized and corresponding nutrition contents were determined.

#### Indirect comparisons between 3D facial shapes and eating behaviors using clustering of the subject groups based on holistic eating behaviors

To visualize the 3D faces corresponding to the holistic nutritional patterns, we subcategorized the participant samples into *k* groups using the k-means clustering method, when the input was a combined vector of nPCs and the sum scores of the eating behavior. In this instance, k-means clustering is a method of dividing *n* observations (n = the number of the participants) into *k* groups, with each observation belonging to the cluster with the closest mean (cluster centroid) that serves as the cluster prototype. k-means clustering starts from randomized initial assignments of the observation and calculation of the cluster centroids. The method then continues iterations to reassign observations and calculate the centroids, so that movement minimizes the variance (squared Euclidean distance) within a cluster. Once no movement of the centroids is observed, the iterations are considered finished. The number of clusters was determined by the elbow method. Each cluster was represented by the cluster centroid of the categorized group (hereafter referred to as the “code”). To visualize the 3D facial shapes for each code, the 3D-averaged and accentuated faces (Supplementary Text S6) for each code were calculated using the aforementioned fitted meshes on the 3D face.

### Calculation of SShD as an index for feminine or masculine facial shapes

The SShD of the individual face was measured by projection of the individual facial configurations onto a male–female axis that were determined a previous study^30^. In short, the male–female axis was calculated from 272 Turkish and Japanese men and women and defined the vector between the average facial configurations of males and females in the facial morphospace using the following equation^31,81^

$$\mathrm{SShD}\left(\overrightarrow{{F}_{i}}\right)=\frac{( \overrightarrow{{F}_{i}}\cdot \overrightarrow{{F}_{(m-f)}})}{{|\overrightarrow{{F}_{(m-f)}}|}^{2}}$$

where $\overrightarrow{{F}_{i}}$ is the vector in the facial morphospace corresponding to an individual face *i*, and $\overrightarrow{{F}_{(m-f)}}$ is the vector between male and female facial configurations (male minus female previously determined^30^. If SShD < − 1, the face is hyperfeminine, and if SShD > 1, the face is hypermasculine. We used mixed samples of Japanese and Turkish individuals to calculate the SShD because we aimed to extract vectors to represent sexual dimorphism with population affinity.

Measures of SShD were mathematically decomposed to allometric and non-allometric components. That is, variations in SShD due to an individual’s size (allometric) and variations that were independent of size (non-allometric) were examined in the overall variation in SShD in each population group using a multivariate regression analysis. CS was used as a measure of an individual’s size.

### Inter-code subject group comparisons

The analysis of variance (ANOVA) and the Tukey–Kramer post-hoc tests were conducted to examine whether or not any nutrient content variable was specific to each code (i.e. pattern) and whether or not any eating behavior major scales were specific to each code.

In addition, the faces were examined by using a total of 17 variables were extracted as inter-landmark distances of the face (|Ac-Ac|, |Zy-Zy|, |Ch-Ch|, |Go-Go|, |Go-Go|/|Zy-Zy|, |N-En|, |N-Sn|, |N-Zy|, |N-Prn|, |N-Ls|, |Sto-Gn|, |Ls-Li|, |Gla-Sn|/|Sn-Gn|, |N-Gn|/|Zy-Zy|, |Gla-ls|/|Zy-Zy|, |Gla-ls|/|Zy-Zy|, |Ps-Pi|; For definition, please see Supplementary Fig. S1 and Supplementary Table S7). Facial size differences between individuals were also standardized by normalizing the values of all linear variables to the distance between right and left exocanthions. ANOVA and the Tukey–Kramer post-hoc tests were conducted to these facial variables in addition to CS, SShD, allometric SShD, non-allometric SShD to examine if there were any differences between the codes.

Further, adjunctive data for health history, which were assumed as relevant to the dietary conditions (i.e. physical activity level, duration of physical exercises, birth weight, 2-year change in weight, sleep duration, and menarche age) were also collected from each participant for inter-code subject group comparisons. Physical body composition (e.g. weight, total fat mass) and adjunctive data for health history were also compared between codes.

*p* values were adjusted for multiple comparisons using the Benjamini–Hochberg method in order to control the false discovery rate^82^. Adjusted values of *p* < 0.05 were significant for all statistical tests.

## Supplementary Information

Supplementary Information.

## Data Availability

The datasets used and/or analysed during the current study available from the corresponding author on reasonable request.
