# Supplementary material for: Influence of the nutritional status on facial morphology in young Japanese women
Source: Sci Rep. 2022 Nov 3;12:18557. doi: 10.1038/s41598-022-21919-5 (PMC9633753; doi:10.1038/s41598-022-21919-5)
Supplement: Supplementary file 1 — Supplementary Information. [file 41598_2022_21919_MOESM1_ESM.docx]

**Supporting information**

**Influence of the nutritional status on facial morphology in young Japanese women**

*Chihiro Tanikawa^1*^, Miki Kurata^3^, Noriko Tanizaki^3^, Mika Takeuchi^3^, Edlira Zere^1^, Keisuke Fukuo^3^, and Kenji Takada^2^*

^1^ Department of Orthodontics and Dentofacial Orthopedics, Osaka University Dental Hospital, Suita, Osaka, Japan

^2^ Center for Advanced Medical Engineering and Informatics, Osaka University, Suita, Osaka, Japan

^3^ Department of Food Sciences and Nutrition, School of Human Environmental Sciences, Mukogawa Women's University, Nishinomiya, Hyogo, Japan

* tanikawa.chihiro.dent@osaka-u.ac.jp and [ctanika@gmail.com](mailto:ctanika@gmail.com)

***Supplementary Text S1. Details of nutrient content in each code***

Supplementary Tables S3 and 4 show statistical comparisons of the three subject groups for the nutrient variables.

The Code 2 subject group (n = 36) exhibited distinct differences from the Code 1 and 3 subject groups. This group was characterized by a significantly greater intake of energy, protein, lipid, retinol, tocopherol, vitamins B2 and B12, niacin, and other minerals. The Code 2 group also showed greater values for nPC 1 than Codes 1 and 3, indicating that Code 2 was a group with a greater total amount of overall nutrition. Code 2 was also characterized by a smaller nPC 3, indicating a greater total intake of energy, lipids, and FAs (negative direction of nPC 3).

The food sources and dietary intake of the women designated as Codes 1 and 3 (n = 45 and n=34, respectively) were characterized by similar variables when compared to the Code 2 subject group, e.g. lower intakes of energy with lower lipids and total FAs, dietary cholesterol, mono- and polyunsaturated FAs, pantothenic acid, tocopherol, minerals, and vitamins B1, B2, and B6. nPC 1 showed the same results, with Codes 1 and 3 showing a lower energy intake than Code 2 (p<0.05). However, Code 3 was exclusively characterized by significantly smaller values of animal protein ratio and fish oil ratio; a greater intake of n-6 polyunsaturated FA, salt, and calcium; and a smaller intake of vitamin D, niacin, vitamin B12, and n-3 polyunsaturated FAs than Code 1.

nPC 4 also showed smaller values in Code 1 than in the other groups, indicating a greater fish oil ratio and greater intake of protein relative to the total energy. In contrast, Code 3 was characterized by greater nPCs 2 and 5 and a smaller nPC 6. This indicates a greater vegetable oil ratio (positive direction of nPC 2), greater n-6/n-3 unsaturated FA ratio (positive direction of nPC 5), greater consumption of calcium and lipids (%energy), and a higher saturated FA ratio to the total FA amount (negative direction of nPC6).

These results indicate that both Codes 1 and 3 were low-energy (lipid) groups, but Code 3 had less animal-related nutrition (e.g. smaller intake of n-3 polyunsaturated FA, vitamin D, vitamin B12, niacin, and animal protein) and greater vegetable-related nutrition (e.g. greater vegetable oil ratio, carbohydrate, n-6 polyunsaturated FA). Thus, Code 3 is considered an imbalanced low-calorie-intake group.

In summary, Code 1 was characterized as a “balanced low-calorie-intake group”, Code 2 was characterized as a “high-calorie intake group”, and Code 3 was characterized as an “imbalanced low-calorie intake group”.

(all: p < 0.05; p values were adjusted for multiple comparisons using the Benjamini-Hochberg method ^1^).

***Supplementary Text S2. Details of eating behavior in each code***

Code 1 was characterized by healthy eating behaviors in all subcategories of questions (i.e. recognition of weight and constitution, emotional eating behavior, eating style, external eating behavior, food preference, regular eating habits), whereas Code 3 was associated with unhealthy eating behaviors (i.e. recognition of weight and constitution, emotional eating behavior), and Code 2 showed unhealthy eating behaviors for all subcategories (Supplementary Table S4).

In details, with regard to the recognition of weight and constitution, the Code 2 and Code 3 groups were sensitive in recognizing body weight and constitution (Q2, Q22, Q42). Especially, subjects comprising Code 2 think gain weight even they did not eat. For the emotional eating behavior, women in the Code 2 and Code 3 groups tended to yield to the temptation to eat more externally, compared to the than Code 1 women, particularly under emotional stress (Q16). With regard to eating style, the Code 2 and Code 3 subject groups reported they ate faster, ate a higher quantity of food (Q55), with less chewing time and in succession (Q25), compared to the Code 1 subject group.

With regard to the external eating behavior, the Codes 2 subject group tended to order, buy and prepare more foods than they can eat (Q28, Q33, Q38). With regard to the sensation of hunger, the Codes 2 subject group overall felt more anxious about their yielding to temptation to eat a lot of food and felt remorse after eating (Q9, Q15). For food preference, the Code 2 subject group had more chances of going out or having food delivered (Q26) and fatty foods (Q43) compared to the Code 1 subject group.

The Code 1 subject group similarly preferred Japanese-style foods to American-style foods, and seldom ate snacks and sweets, compared to the Code 2 subject group (Q11, Q52). With regard to the regularity of eating habits, the Code 2 subject group took dinner late at night (Q4), ate at irregular meal times (Q21, Q37), ate heavy dinner (Q35; p < 0.01). The Code 3 subject group often skipped breakfast (Q48, all, p < 0.01).

In summary, both Codes 2 and 3 subject groups showed unhealthy eating behaviors with more emotional eating behavior and faster eating styles and felt easily gain weight, but Code 3 is a subject group who healthier sensation of hunger and food preference (e.g., less lipid-included foods) when compared with Code 2. Code 1 is a subject group who had healthiest eating behaviors.

A Cronbach’s alpha of 0.92 for all questions of self-reported survey indicated high internal consistency.

***Supplementary Text S3. Soft-tissue facial measurements that were significantly different among the three subject groups***

A landmark-based analysis showed that the zygomatic width was greater in Code 2 than in Code 1 (|Zy-Zy|, p <0.05). Code 2 also showed a lower vertical position of the nasal bridge relative to the eyes than Code 1 and Code 3 subjects (|N-En|, p < 0.05), indicating a low and depressed nasal bridge ^2^. A low nasal bridge is generally related to a short nose, flat nasal bridge, and flattened midface ^3^.

Code 3 showed a larger mandibular height than Codes 1 and 2 (|Sto-Gn| and |Gla-Sn|/|Sn-Gn|, p<0.05). Code 3 was also characterized by a lower eye height than Codes 2 and 3.

sPC 1 also showed greater values in Code 2 than in Code 1, indicating that Code 2 was associated with a greater facial width, midfacial retrusion, protrusion of the chin, and anterior divergent face (p<0.05).

***Supplementary Text S4.*** ***Other health history of the participants***

To examine whether any health history of the participants are specific to each dietary intake condition, a total of 43 participants out of the 151 were selected by a random sampling method, and physical activity level (PAL) was analyzed by using the aforementioned software (Eiyo-kun; Kenpaku, Inc.). The PAL was defined as the total energy expenditure (kcal/day) divided by the basal metabolic rate (kcal/day), based on the self-reported data. In addition, five parameters (i.e. duration of physical exercises, birth weight, 2-year change in weight, sleep duration, and menarche age) were also examined. Sleep duration was examined because decreased sleep duration has been reported to be associated with an increase in body weight and adiposity. As a result, there were no significant differences between the code groups in PAL, duration of physical exercises, birth weight, 2-year change in weight, sleep duration, or menarche age (ANOVA, *p* > 0.05, Supplementary Table S9).

Furthermore, the percentage of the participants living with their parents in each group was 83.3%, 86.6%, and 66.7% in Code 1, Code 2, and Code 3, respectively, which shows no significant differences (chi-square test of independence to test for equality of proportions, *p* > 0.05).

***Supplementary Text S5.*** ***Relationships between body compositions and nutritional intakes or eating behaviors***

The muscle mass was found to be significantly correlated with energy (r = 0.19, p = 0.04), sodium (0.21, p = 0.02), and beta tocopherol (r = 0.21, p = 0.03), while the body weight, fat mass, and body mass index (BMI) showed no significant correlations with any nutritional intakes. With regard to the total scores for the eating behaviours, body weight (r =0.32, p <0.001), muscle mass (r =0.21, p<0.021), fat mass (r=0.28, p<0.002), and BMI (r=0.30, p =0.02) showed significant correlations.

***Supplementary Text S6. Calculation of the averaged faces and accentuated average faces***

For each participant, a wire mesh, based on the assignment of landmarks to each 3D facial image, was fitted using software (HBM-Rugle; Medic Engineering Co., Kyoto, Japan). This method generated 6,017 points on the wire mesh (i.e. the nodes of the fitted mesh) for each facial expression. The arithmetic mean of the coordinate values and the color values of each corresponding point on the wire mesh were computed and used to generate the 3D averaged facial images for each subject group.

To quantitatively facilitate the instantaneous and intuitive understanding of differences among the codes in the facial forms, accentuated averaged faces AccA_(Code 1)_, AccA_(Code 2)_, and AccA_(Code 3)_ were calculated for the Code 1, Code 2, and Code 3 groups, respectively, to highlight the differences between the two subject groups, in which

AccA_(Code 1)_ = A_(All)_ + *w* (A_(Code 1)_ - A_(All)_) (*w* = 5)

AccA_(Code 2)_ = A_(All)_ + *w* (A_(Code 2)_ - A_(All)_) (*w* = 5)

AccA_(Code 3)_ = A_(All)_ + *w* (A_(Code 3)_ - A_(All)_) (*w* = 5)

and A_(Code 1)_, A_(Code 2)_, A_(Code 3)_, and A_(All)_ are the arithmetic means of the coordinate values for the Code 1, Code 2, and Code 3, respectively, and *w* is the weight value.

***Supplementary Table S1. Results of a step-wise regression analysis for each dependent variable (nPCs 4 and 6, eating behaviour score, body weight, muscle mass, body fat mass, and body mass index).***

*The coefficient of determination, F statistics, and p-value when facial sPCs were used as independent variables, and nPCs and behaviour scores, physical variables were used as dependent variables in the regression analysis.*

*MSE, mean square error; R^2^, coefficient of determination, df, degree of freedom; sPC, shape principal component; nPC, nutrition principal component.*

| Dependent variables | MSE | R^2^ | F statistics | p value | Independent variables | Estimate | MSE | t statistics | p value |
| --- | --- | --- | --- | --- | --- | --- | --- | --- | --- |
| nPC4 | 0.98 | 0.06 | 6.37 | 0.01 | (Intercept) | 0 | 0.09 | 0.00 | 1 |
|  |  |  |  |  | sPC1 | 0.24 | 0.09 | 2.52 | 0.01 |
| nPC6 | 0.96 | 0.09 | 5.54 | 0.01 | (Intercept) | 0.00 | 0.09 | 0.00 | 1 |
|  |  |  |  |  | sPC1 | 0.21 | 0.09 | 2.31 | 0.02 |
|  |  |  |  |  | sPC4 | 0.22 | 0.09 | 2.40 | 0.02 |
| Eating behavior score | 0.99 | 0.04 | 4.35 | 0.04 | (Intercept) | 0.00 | 0.09 | 0.00 | 1 |
|  |  |  |  |  | sPC1 | 0.20 | 0.09 | 2.09 | 0.04 |
| Total body weight | 0.94 | 0.12 | 14.4 | 0.00 | (Intercept) | 0.00 | 0.09 | 0.00 | 1 |
|  |  |  |  |  | sPC5 | 0.34 | 0.09 | 3.79 | 0 |
| Total muscle mass | 0.96 | 0.08 | 9.65 | 0.00 | (Intercept) | 0.00 | 0.09 | 0.00 | 1 |
|  |  |  |  |  | sPC5 | 0.29 | 0.09 | 3.11 | 0 |
| Total body fat mass | 0.95 | 0.11 | 6.46 | 0.00 | (Intercept) | 0.00 | 0.09 | 0.00 | 1 |
|  |  |  |  |  | sPC5 | 0.25 | 0.09 | 2.7 | 0.01 |
|  |  |  |  |  | sPC7 | 0.22 | 0.09 | 2.37 | 0.02 |
| Body mass index | 0.94 | 0.14 | 8.66 | 0.00 | (Intercept) | 0.00 | 0.09 | 0.00 | 1 |
|  |  |  |  |  | sPC1 | 0.23 | 0.09 | 2.57 | 0.01 |
|  |  |  |  |  | sPC5 | 0.30 | 0.09 | 3.28 | 0 |

***Supplementary Table S2. Results of a MANCOVA for dependent variables (nPCs 4 and 6, eating behaviour score, body weight, muscle mass, body fat mass, and body mass index; and independent variables sPCs 1, 4, 5 and 7, which were significant in the step-wise analysis for each regression)***

*R^2^, coefficient of determination; SS, Sum of squares; df, degree of freedom; MSE, mean square error; sPC, shape principal component; nPC, nutrition principal component; MANCOVA, multivariate analysis of covariance*

| Dependent variable | R^2^ | Type III SS | df | MSE | F statistics | p value |  | Partial η^2^ |
| --- | --- | --- | --- | --- | --- | --- | --- | --- |
| nPC4 | 0.08 | 9.33 | 4.00 | 2.33 | 2.38 | 0.06 | . | 0.08 |
| nPC6 | 0.10 | 11.40 | 4.00 | 2.85 | 3.00 | 0.02 | * | 0.10 |
| Total score for eating behaviors | 0.05 | 4.89 | 4.00 | 1.22 | 1.23 | 0.30 |  | 0.05 |
| Body weight | 0.14 | 15.61 | 4.00 | 3.91 | 4.29 | 0.00 | ** | 0.14 |
| Total muscle mass | 0.10 | 11.16 | 4.00 | 2.79 | 2.88 | 0.03 | * | 0.10 |
| Total body fat mass | 0.12 | 13.02 | 4.00 | 3.25 | 3.69 | 0.01 | * | 0.12 |
| Body mass index | 0.16 | 17.48 | 4.00 | 4.37 | 4.97 | 0.00 | ** | 0.16 |

., p<0.1; *, p<0.05; **, P <0.01

***Supplementary Table S3. Comparisons of three subject groups (i.e. codes) determined by vector quantization with the nutrient content variables employed as the feature vector elements***

|  | Code 1 (n = 45) | | Code 2 (n = 36) | | Code 3 (n = 34) | | *p*-value | | F-value | Effect size (ɳ^2^) | Recommended intake ^d^ |
| --- | --- | --- | --- | --- | --- | --- | --- | --- | --- | --- | --- |
|  | Mean | S.D. | Mean | S.D. | Mean | S.D. |  |  |  |  |  |
| Energy (kcal) | 1582.0 | 329.7 | 1933.4 | 277.5 | 1653.4 | 255.4 | 0.00 | * | 15.4 | 0.22 | 1950 |
| Grain (%E) | 37.7 | 6.1 | 33.0 | 7.3 | 36.5 | 7.9 | 0.01 | * | 4.6 | 0.08 | NA |
| Water (g) | 732.1 | 188.2 | 881.4 | 183.5 | 775.1 | 184.5 | 0.00 | * | 6.7 | 0.11 | NA |
| Protein (g) | 57.0 | 10.9 | 68.1 | 12.6 | 55.1 | 11.5 | 0.00 | * | 13.4 | 0.19 | 50 |
| Protein (%E) | 14.5 | 1.5 | 14.1 | 1.7 | 13.3 | 1.6 | 0.00 | * | 6.2 | 0.10 | NA |
| Animal protein ratio (% total protein) | 57.7 | 6.1 | 57.8 | 7.4 | 49.9 | 8.2 | 0.00 | * | 14.6 | 0.21 | NA |
| Lipid (g) | 56.5 | 15.9 | 70.6 | 13.9 | 57.7 | 11.5 | 0.00 | * | 11.5 | 0.17 | NA |
| Lipid (%E) | 32.1 | 3.9 | 32.8 | 4.0 | 31.3 | 3.7 | 0.27 | NS | 1.3 | 0.02 | 20-29 |
| Fatty acid total amount (g) | 49.3 | 14.3 | 61.2 | 12.3 | 50.0 | 10.3 | 0.00 | * | 10.6 | 0.16 | NA |
| Saturated FA (g) | 18.2 | 4.8 | 23.2 | 5.0 | 19.2 | 4.4 | 0.00 | * | 11.8 | 0.17 | 4.5-7.0 |
| Cholesterol (mg) | 296.5 | 73.6 | 383.1 | 83.0 | 291.8 | 62.9 | 0.00 | * | 17.8 | 0.24 | 600 |
| Monounsaturated FA (g) | 20.4 | 5.9 | 25.1 | 5.3 | 19.8 | 4.3 | 0.00 | * | 10.8 | 0.16 | NA |
| Polyunsaturated FA (g) | 10.7 | 4.5 | 12.9 | 3.0 | 10.9 | 2.6 | 0.01 | * | 4.6 | 0.08 | NA |
| n-3 Polyunsaturated FA (g) | 1.8 | 0.7 | 2.2 | 0.6 | 1.6 | 0.5 | 0.00 | * | 7.3 | 0.12 | ≥1.8 |
| n-6 Polyunsaturated FA (g) | 8.9 | 3.8 | 10.7 | 2.4 | 9.2 | 2.1 | 0.02 | * | 4.1 | 0.07 | 9 |
| n-6/n-3 FA ratio | 5.3 | 1.8 | 5.0 | 0.7 | 5.8 | 0.8 | 0.05 | NS | 3.1 | 0.05 | NA |
| Saturated FA ratio ^a^ | 3.7 | 0.4 | 3.8 | 0.3 | 3.8 | 0.4 | 0.37 | NS | 1.0 | 0.02 | NA |
| Monounsaturated FA ratio ^a^ | 4.1 | 0.2 | 4.1 | 0.2 | 4.0 | 0.2 | 0.00 | * | 6.2 | 0.10 | NA |
| Polyunsaturated FA ratio ^a^ | 2.1 | 0.4 | 2.1 | 0.3 | 2.2 | 0.3 | 0.66 | NS | 0.4 | 0.01 | NA |
| Animal oil ratio ^b^ | 5.3 | 1.1 | 5.0 | 1.0 | 4.9 | 0.8 | 0.15 | NS | 1.9 | 0.03 | NA |
| Vegetable oil ratio ^b^ | 4.2 | 1.0 | 4.5 | 0.9 | 4.8 | 0.9 | 0.02 | * | 4.3 | 0.07 | NA |
| Fish oil ratio ^b^ | 0.6 | 0.2 | 0.6 | 0.2 | 0.3 | 0.2 | 0.00 | * | 13.5 | 0.20 | NA |
| Carbohydrate (g) | 203.7 | 41.3 | 243.0 | 40.3 | 220.2 | 35.1 | 0.00 | * | 10.0 | 0.15 | NA |
| Carbohydrate (%E) | 53.4 | 4.7 | 53.1 | 5.2 | 55.4 | 4.7 | 0.09 | NS | 2.5 | 0.04 | 50-69 |
| Deep-yellow-vegetable ratio ^c^ | 37.5 | 9.8 | 34.8 | 10.1 | 40.3 | 9.1 | 0.06 | NS | 2.9 | 0.05 | NA |
| Dietary fiber water solubility (g) | 2.4 | 0.7 | 2.7 | 0.8 | 2.6 | 0.6 | 0.15 | NS | 1.9 | 0.03 | NA |
| Dietary fiber insolubility (g) | 7.5 | 2.2 | 8.0 | 2.1 | 7.8 | 2.1 | 0.58 | NS | 0.5 | 0.01 | NA |
| Dietary fiber total amount (g) | 10.3 | 3.0 | 11.3 | 3.0 | 10.9 | 2.9 | 0.38 | NS | 1.0 | 0.02 | 17 |
| Retinol (μg) | 195.8 | 51.2 | 251.6 | 58.2 | 207.3 | 44.3 | 0.00 | * | 12.5 | 0.18 | NA |
| Alpha carotene (μg) | 470.4 | 217.6 | 415.5 | 223.0 | 509.7 | 226.4 | 0.21 | NS | 1.6 | 0.03 | NA |
| Beta carotene (μg) | 2847.8 | 1291.4 | 2536.8 | 1315.2 | 3059.1 | 1350.3 | 0.25 | NS | 1.4 | 0.02 | NA |
| Cryptoxanthin (μg) | 492.7 | 336.0 | 405.4 | 326.4 | 326.4 | 352.8 | 0.10 | NS | 2.4 | 0.04 | NA |
| Beta carotene equivalent (μg) | 3337.6 | 1465.9 | 2959.8 | 1470.4 | 3489.0 | 1569.4 | 0.31 | NS | 1.2 | 0.02 | NA |
| Retinol equivalent (μg) | 477.3 | 142.7 | 508.8 | 155.5 | 503.9 | 151.4 | 0.59 | NS | 0.5 | 0.01 | 650 |
| Vitamin D (μg) | 4.6 | 1.5 | 6.3 | 2.3 | 3.5 | 1.7 | 0.00 | * | 19.5 | 0.26 | 5.5 |
| Alpha tocopherol (mg) | 5.3 | 1.7 | 6.3 | 1.5 | 5.4 | 1.2 | 0.01 | * | 4.7 | 0.08 | 6.5 |
| Beta tocopherol (mg) | 0.3 | 0.1 | 0.4 | 0.1 | 0.3 | 0.1 | 0.04 | NS | 3.5 | 0.06 | NA |
| Gamma tocopherol (mg) | 9.7 | 5.2 | 11.9 | 3.4 | 10.5 | 2.7 | 0.05 | NS | 3.1 | 0.05 | NA |
| Delta tocopherol (mg) | 2.5 | 1.4 | 3.0 | 0.9 | 2.8 | 0.9 | 0.09 | NS | 2.4 | 0.04 | NA |
| Tocopherol equivalent (mg) | 6.5 | 2.2 | 7.7 | 1.8 | 6.6 | 1.4 | 0.01 | * | 4.5 | 0.08 | NA |
| Vitamin K (μg) | 169.4 | 65.2 | 173.5 | 61.9 | 182.9 | 64.5 | 0.64 | NS | 0.4 | 0.01 | 60 |
| Vitamin B1 (mg) | 0.8 | 0.2 | 0.9 | 0.2 | 0.8 | 0.2 | 0.00 | * | 7.5 | 0.12 | 1.1 |
| Vitamin B2 (mg) | 0.9 | 0.2 | 1.2 | 0.2 | 1.0 | 0.2 | 0.00 | * | 13.5 | 0.19 | 1.2 |
| Niacin (mg) | 12.1 | 2.6 | 14.6 | 3.8 | 10.3 | 2.8 | 0.00 | * | 17.1 | 0.23 | 11 |
| Vitamin B6 (mg) | 0.9 | 0.2 | 1.0 | 0.3 | 0.8 | 0.2 | 0.00 | * | 6.6 | 0.11 | 1.1 |
| Vitamin B12 (μg) | 4.9 | 1.3 | 6.3 | 2.1 | 3.7 | 1.7 | 0.00 | * | 20.6 | 0.27 | 2.4 |
| Folic acid (μg) | 224.8 | 66.2 | 236.4 | 68.9 | 232.5 | 70.5 | 0.73 | NS | 0.3 | 0.01 | 240 |
| Pantothenic acid (mg) | 4.7 | 1.0 | 5.6 | 1.0 | 4.8 | 0.9 | 0.00 | * | 9.6 | 0.15 | 5 |
| Vitamin C (mg) | 77.9 | 31.1 | 75.2 | 29.1 | 70.6 | 34.3 | 0.60 | NS | 0.5 | 0.01 | 100 |
| Mineral (g) | 12.1 | 3.0 | 15.5 | 3.1 | 12.9 | 3.2 | 0.00 | * | 12.5 | 0.18 | NA |
| Sodium (mg) | 2576.0 | 767.2 | 3503.0 | 830.2 | 2792.3 | 931.0 | 0.00 | * | 12.9 | 0.19 | NA |
| Salt (g) | 6.5 | 1.9 | 8.9 | 2.1 | 7.1 | 2.4 | 0.00 | * | 13.1 | 0.19 | ＜7.5 |
| Potassium (mg) | 1833.0 | 477.1 | 2127.0 | 489.6 | 1872.7 | 483.3 | 0.02 | * | 4.1 | 0.07 | 2000 |
| Calcium (mg) | 432.8 | 126.3 | 551.1 | 141.1 | 515.8 | 173.3 | 0.00 | * | 7.1 | 0.11 | 650 |
| Magnesium (mg) | 181.8 | 47.2 | 216.8 | 46.6 | 189.5 | 47.3 | 0.00 | * | 5.9 | 0.10 | 270 |
| Phosphorus (mg) | 831.8 | 173.2 | 1011.8 | 180.4 | 851.2 | 188.1 | 0.00 | * | 11.4 | 0.17 | 900 |
| Iron (mg) | 5.8 | 1.5 | 6.9 | 1.7 | 6.0 | 1.5 | 0.00 | * | 5.8 | 0.09 | 10.5 |
| Zinc (mg) | 7.0 | 1.4 | 8.1 | 1.4 | 6.8 | 1.4 | 0.00 | * | 8.5 | 0.13 | 9 |
| Copper (mg) | 0.8 | 0.2 | 1.0 | 0.2 | 0.9 | 0.2 | 0.02 | * | 4.1 | 0.07 | 0.7 |
| Manganese (mg) | 2.0 | 0.5 | 2.2 | 0.4 | 2.0 | 0.4 | 0.22 | NS | 1.5 | 0.03 | 3.5 |

1. Ratio to the total fatty acids
2. Ratio to the sum of animal oil, vegetable oil, and fish oil
3. Ratio to the sum of vegetables
4. Japanese Female Nutrient Data (18–29 y) from the Ministry of Health, Labour and Welfare, Tokyo, Japan 2015; <http://www.mhlw.go.jp/file/04-Houdouhappyou-10904750-Kenkoukyoku-Gantaisakukenkouzoushinka/0000041955.pdf>; Accessed 5/7/2021; in Japanese. A

* *p* < 0.05 (p values were adjusted for multiple comparisons using the Benjamini-Hochberg method ^1^.)

FA, fatty acid; NS, not significant; NA, not available; S.D., standard deviation

***Supplementary Table S4. Comparisons of three subject groups (Codes 1–3) for the nutritional principal components (nPCs) 1, 2, …,7.***

|  | Code 1  (n = 45) | | Code 2  (n = 36) | | Code 3  (n = 34) | | p-value | | F-value | Effect size (ɳ^2^) |
| --- | --- | --- | --- | --- | --- | --- | --- | --- | --- | --- |
|  | Mean | S.D. | Mean | S.D. | Mean | S.D. |  |  |  |  |
| nPC1 | -0.25 | 1.02 | 0.50 | 0.93 | -0.19 | 0.86 | 0.00 | * | 7.32 | 0.12 |
| nPC2 | -0.13 | 1.00 | -0.22 | 1.04 | 0.40 | 0.85 | 0.02 | * | 4.15 | 0.07 |
| nPC3 | 0.30 | 0.99 | -0.51 | 0.81 | 0.14 | 1.01 | 0.00 | * | 8.04 | 0.13 |
| nPC4 | -0.37 | 0.93 | -0.02 | 1.06 | 0.52 | 0.79 | 0.00 | * | 8.79 | 0.14 |
| nPC5 | 0.05 | 1.18 | -0.45 | 0.75 | 0.41 | 0.77 | 0.00 | * | 7.26 | 0.12 |
| nPC6 | 0.12 | 0.77 | 0.28 | 1.00 | -0.46 | 1.13 | 0.00 | * | 5.76 | 0.09 |
| nPC7 | 0.13 | 1.04 | -0.09 | 0.99 | -0.08 | 0.98 | 0.54 |  | 0.61 | 0.01 |

***Supplementary Table S5. Comparisons of three subject groups (Codes 1–3) for the dietary habit variables (the questionnaire consisted of 55 questions on seven major scales).***

|  | Code 1  (n = 45) | | Code 2  (n = 36) | | Code 3  (n = 34) | | p-value | | F-value | Effect size (ɳ^2^) |
| --- | --- | --- | --- | --- | --- | --- | --- | --- | --- | --- |
|  | Mean | S.D. | Mean | S.D. | Mean | S.D. |  | |  |  |
| Q (Recognition of weight and constitution) | 2.1 | 0.6 | 2.8 | 0.5 | 2.8 | 0.4 | 0.00 | * | 27.41 | 0.33 |
| Q2 Do you think you gain weight because you like eating sweets? | 2.2 | 1.0 | 3.2 | 0.8 | 3.2 | 0.8 | 0.00 | * | 16.38 | 0.23 |
| Q6 Do you think you gain weight because you lie down immediately after eating? | 2.2 | 1.1 | 2.6 | 1.0 | 2.8 | 0.9 | 0.02 | * | 4.22 | 0.07 |
| Q10 Do you think you eat a lot even when you have a cold? | 2.4 | 1.1 | 3.0 | 1.1 | 2.8 | 1.0 | 0.03 | * | 3.60 | 0.06 |
| Q22 Do you think you gain weight even after you drink water? | 1.2 | 0.4 | 1.8 | 0.9 | 1.8 | 0.8 | 0.00 | * | 10.53 | 0.16 |
| Q36 Do you think you gain weight because of lack of exercise? | 2.9 | 1.0 | 3.4 | 0.8 | 3.4 | 0.8 | 0.01 | * | 4.41 | 0.07 |
| Q42 Do you think it is easier for you than others to gain weight? | 1.8 | 1.0 | 2.8 | 0.9 | 2.9 | 0.9 | 0.00 | * | 16.98 | 0.23 |
| Q51 Is it difficult to lose weight even you are trying not to eat so much? | 1.5 | 0.8 | 1.7 | 0.8 | 2.0 | 1.0 | 0.05 |  | 2.99 | 0.05 |
| Q (External eating behavior) | 1.9 | 0.39 | 2.7 | 0.6 | 2.4 | 0.4 | 0.00 | * | 31.58 | 0.36 |
| Q13 If food smells and looks good, do you eat more than usual? | 2.9 | 1.0 | 3.6 | 0.7 | 3.3 | 0.9 | 0.00 | * | 6.03 | 0.10 |
| Q17 Do you complain if the number of dishes served is few? | 1.8 | 0.9 | 2.2 | 1.1 | 1.9 | 1.0 | 0.18 |  | 1.72 | 0.03 |
| Q24 If you see others eating, do you also have the desire to eat? | 2.3 | 0.9 | 3.4 | 0.7 | 3.2 | 0.7 | 0.00 | * | 23.07 | 0.29 |
| Q28 Do you tend to order more food than you can eat when you go to a restaurant? | 1.2 | 0.6 | 2.3 | 1.1 | 1.8 | 0.7 | 0.00 | * | 16.19 | 0.22 |
| Q33 Do you tend to buy more food than needed? | 1.6 | 0.7 | 2.5 | 1.0 | 2.1 | 0.9 | 0.00 | * | 10.01 | 0.15 |
| Q38 Do you tend to prepare more dishes than you can eat? | 1.4 | 0.6 | 2.3 | 1.1 | 1.8 | 0.8 | 0.00 | * | 9.98 | 0.15 |
| Q44 If you walk past the supermarket, do you have the desire to buy something delicious? | 2.3 | 1.0 | 3.0 | 1.1 | 2.6 | 0.9 | 0.01 | * | 4.61 | 0.08 |
| Q50 Do you tend to eat with other people? | 1.4 | 0.6 | 2.1 | 1.0 | 1.8 | 1.0 | 0.00 | * | 6.36 | 0.10 |
| Q12 Do you eat all dishes because you do not like to leave food? | 2.5 | 1.0 | 3.3 | 0.8 | 3.2 | 0.9 | 0.00 | * | 9.66 | 0.15 |
| Q (Emotional eating behavior) | 1.7 | 0.5 | 2.5 | 0.6 | 2.4 | 0.5 | 0.00 | * | 28.89 | 0.34 |
| Q5 Do you feel uneasy if you do not have enough food in the refrigerator? | 1.3 | 0.6 | 1.8 | 1.0 | 2.0 | 1.0 | 0.00 | * | 5.81 | 0.09 |
| Q16 Do you have the desire to eat when you are irritated? | 1.9 | 0.8 | 3.1 | 0.8 | 3.0 | 0.9 | 0.00 | * | 24.80 | 0.31 |
| Q23 Do you keep something to eat around you? | 1.7 | 0.8 | 2.4 | 1.0 | 2.1 | 0.8 | 0.00 | * | 5.80 | 0.09 |
| Q31 Do you have a desire to eat when you have nothing to do? | 1.9 | 0.7 | 2.9 | 0.9 | 2.6 | 0.8 | 0.00 | * | 18.30 | 0.25 |
| Q34 Do you eat snacks or fruits if you find them around you? | 2.4 | 0.8 | 3.4 | 0.8 | 3.0 | 0.7 | 0.00 | * | 14.04 | 0.20 |

* *p* < 0.05 (*p* values were adjusted for multiple comparisons using the Benjamini-Hochberg method. ^1^)

^a^ Not included in the major scale total scores.

S.D., standard deviation.

***Supplementary Table S5 (Cont'd)***

|  | Code 1  (n = 45) | | Code 2  (n =36) | | Code 3  (n = 34) | | p-value | | F-value | | Effect size (ɳ^2^) | |
| --- | --- | --- | --- | --- | --- | --- | --- | --- | --- | --- | --- | --- |
|  | Mean | S.D. | Mean | S.D. | Mean | S.D. |  | | |  | |  |
| Q (Sense of hunger) | 1.7 | 0.3 | 2.4 | 0.41 | 2.3 | 0.4 | 0.00 | * | | 34.63 | | 0.38 |
| Q9 Do you get irritated when you feel hungry? | 1.9 | 0.9 | 2.9 | 0.9 | 2.6 | 0.9 | 0.00 | * | | 13.80 | | 0.20 |
| Q15 Do you feel unsatisfied until you eat full? | 2.2 | 0.9 | 3.2 | 0.8 | 2.8 | 0.9 | 0.00 | * | | 15.06 | | 0.21 |
| Q32 Do you often regret because you have eaten a lot of food? | 2.6 | 1.0 | 3.3 | 0.8 | 3.4 | 0.8 | 0.00 | * | | 10.46 | | 0.16 |
| Q39 Can you not sleep once you realize that you are hungry? | 1.3 | 0.7 | 2.0 | 1.0 | 1.8 | 0.9 | 0.00 | * | | 7.01 | | 0.11 |
| Q49 Is it difficult to feel hungry or not? | 1.1 | 0.5 | 1.4 | 0.7 | 1.3 | 0.6 | 0.13 |  | | 2.08 | | 0.04 |
| Q53 Do you usually feel full before you eat? | 1.4 | 0.6 | 1.5 | 0.7 | 1.6 | 0.8 | 0.31 |  | | 1.17 | | 0.02 |
| Q45 Do you tend to think about the next meal just after eating? | 1.5 | 0.7 | 2.6 | 1.1 | 2.2 | 1.0 | 0.00 | * | | 12.82 | | 0.19 |
| Q (Eating style) | 1.8 | 0.57 | 2.7 | 0.71 | 2.2 | 0.7 | 0.00 | * | | 19.34 | | 0.26 |
| Q1 Do you eat fast? | 1.9 | 1.0 | 2.6 | 1.1 | 2.1 | 0.8 | 0.01 | * | | 5.48 | | 0.09 |
| Q8 Are you known to eat a lot of food? | 2.0 | 0.9 | 3.3 | 0.8 | 2.4 | 0.9 | 0.00 | * | | 18.77 | | 0.25 |
| Q25 Do you eat without chewing several times? | 1.9 | 0.9 | 2.7 | 1.0 | 2.4 | 1.0 | 0.00 | * | | 7.14 | | 0.11 |
| Q41 Do you eat with your mouth full? | 1.2 | 0.6 | 2.3 | 1.1 | 1.8 | 1.0 | 0.00 | * | | 12.69 | | 0.19 |
| Q55 Do you eat foods one after another? | 1.7 | 0.8 | 2.6 | 1.1 | 2.2 | 1.2 | 0.00 | * | | 8.05 | | 0.13 |
| Q (Food preference) | 1.9 | 0.5 | 2.5 | 0.6 | 2.3 | 0.4 | 0.00 | * | | 15.88 | | 0.22 |
| Q3 Do you often go to convenience stores? | 1.9 | 0.9 | 2.6 | 1.1 | 2.5 | 1.0 | 0.01 | * | | 4.76 | | 0.08 |
| Q19 Do you like noodles? | 2.6 | 1.1 | 3.2 | 0.8 | 2.8 | 0.9 | 0.01 | * | | 5.21 | | 0.09 |
| Q26 Do you often go out or have delivery? | 1.7 | 0.8 | 2.4 | 1.1 | 2.2 | 1.0 | 0.01 | * | | 5.55 | | 0.09 |
| Q30 Do you often eat fast foods? | 1.6 | 0.7 | 1.8 | 0.9 | 1.6 | 0.7 | 0.33 |  | | 1.13 | | 0.02 |
| Q40 Do you often eat snack bread? | 1.8 | 0.9 | 2.1 | 1.2 | 2.2 | 1.0 | 0.17 |  | | 1.79 | | 0.03 |
| Q43 Do you like fatty foods? | 1.8 | 0.7 | 2.7 | 1.0 | 2.5 | 1.0 | 0.00 | * | | 11.45 | | 0.17 |
| Q54 Do you like meat? | 2.1 | 0.9 | 3.0 | 0.9 | 2.6 | 1.0 | 0.00 | * | | 9.66 | | 0.15 |
| Q7 Do you often go drinking? | 1.6 | 0.8 | 2.1 | 1.0 | 1.8 | 0.9 | 0.09 |  | | 2.45 | | 0.04 |
| Q11 Do you often eat snacks? | 1.5 | 0.7 | 2.1 | 0.9 | 1.9 | 0.9 | 0.01 | * | | 4.43 | | 0.07 |
| Q14 Do you like salty taste? | 2.1 | 0.9 | 2.6 | 1.1 | 2.2 | 0.9 | 0.03 | * | | 3.72 | | 0.06 |
| Q29 Do you prefer Western-style foods to Japanese-style foods? | 2.2 | 0.9 | 2.8 | 0.8 | 2.3 | 0.8 | 0.01 | * | | 4.45 | | 0.07 |
| Q46 Do you often drink beer or alcohol? | 1.4 | 0.7 | 1.9 | 1.3 | 1.6 | 0.9 | 0.11 |  | | 2.26 | | 0.04 |
| Q52 Do you like sweets? | 2.3 | 1.1 | 3.0 | 1.0 | 2.9 | 0.8 | 0.00 | * | | 6.55 | | 0.11 |
| Q (Regularity of eating habits) | 2.0 | 0.4 | 2.7 | 0.5 | 2.6 | 0.5 | 0.00 | * | | 24.26 | | 0.30 |
| Q4. Is your dinnertime too late at night? | 1.5 | 0.6 | 2.3 | 1.0 | 1.9 | 0.8 | 0.00 | * | | 9.44 | | 0.14 |
| Q18. Do you tend to stay awake at midnight and have difficulty getting up in the morning? | 2.3 | 1.2 | 2.7 | 1.2 | 2.7 | 1.2 | 0.30 |  | | 1.23 | | 0.02 |
| Q20. Do you gain body weight during holidays? | 2.3 | 1.0 | 3.0 | 0.8 | 2.9 | 0.8 | 0.00 | * | | 6.86 | | 0.11 |
| Q21. Do you often eat between meals? | 2.3 | 1.0 | 3.2 | 0.7 | 3.1 | 0.9 | 0.00 | * | | 12.81 | | 0.19 |
| Q27. Are your meal times irregular? | 1.8 | 0.7 | 2.9 | 1.0 | 2.6 | 1.0 | 0.00 | * | | 16.22 | | 0.23 |
| Q35. Is dinner your heaviest meal? | 2.6 | 0.9 | 3.1 | 1.0 | 3.0 | 0.9 | 0.05 |  | | 3.15 | | 0.05 |
| Q37. Do you eat dinner at or after midnight? | 2.1 | 0.7 | 2.9 | 0.9 | 2.8 | 1.1 | 0.00 | * | | 9.66 | | 0.15 |
| Q48. Do you often skip breakfast? | 1.3 | 0.7 | 1.4 | 0.8 | 1.6 | 0.8 | 0.29 |  | | 1.27 | | 0.02 |
| Q47. Do you feel that you do not have enough time to eat? ^a^ | 1.3 | 0.5 | 1.8 | 0.8 | 1.8 | 0.8 | 0.00 | * | | 6.46 | | 0.10 |
| Total score for eating behaviors (Sum of scores) | 13.2 | 1.8 | 18.3 | 2.6 | 17.0 | 1.8 | 0.00 | * | | 67.76 | | 0.55 |

***Supplementary Table S6. Comparisons of three subject groups (Codes 1–3) for the nutritional principal components (nPCs) 1, 2, …,7.***

|  | Code 1  (n = 41) | | Code 2  (n = 35) | | Code 3  (n = 33) | | p-value | | F-value | Effect size (ɳ^2^) |
| --- | --- | --- | --- | --- | --- | --- | --- | --- | --- | --- |
|  | Mean | S.D. | Mean | S.D. | Mean | S.D. |  |  |  |  |
| sPC1 | -0.31 | 0.95 | 0.23 | 0.99 | 0.15 | 1.00 | 0.04 | * | 3.45 | 0.06 |
| sPC2 | -0.21 | 1.05 | 0.16 | 1.06 | 0.09 | 0.84 | 0.23 |  | 1.48 | 0.03 |
| sPC3 | -0.13 | 1.14 | -0.01 | 0.89 | 0.18 | 0.93 | 0.42 |  | 0.87 | 0.02 |
| sPC4 | 0.15 | 1.06 | -0.11 | 1.04 | -0.07 | 0.88 | 0.46 |  | 0.78 | 0.01 |
| sPC5 | -0.11 | 1.01 | -0.06 | 0.89 | 0.20 | 1.09 | 0.39 |  | 0.96 | 0.02 |
| sPC6 | -0.02 | 0.97 | -0.01 | 0.95 | 0.04 | 1.11 | 0.96 |  | 0.04 | 0.00 |
| sPC7 | -0.05 | 0.94 | 0.15 | 1.00 | -0.10 | 1.08 | 0.57 |  | 0.57 | 0.01 |
| sPC8 | 0.11 | 1.11 | -0.14 | 0.97 | 0.01 | 0.90 | 0.56 |  | 0.58 | 0.01 |

***Supplementary Table S7. Comparisons of three subject groups (Codes 1–3) for variables determined for the three-dimensional facial configuration data.***

| Variable | Representation | Code 1  Balanced diet group  (n = 41) | | Code 2  High-calorie-intake group  (n = 35) | | Code 3  Imbalanced diet with low calories group  (n = 33) | | p-value | | F-value | |  | Effect size (ɳ^2^) |  |
| --- | --- | --- | --- | --- | --- | --- | --- | --- | --- | --- | --- | --- | --- | --- |
|  |  | Mean | S.D. | Mean | S.D. | Mean | S.D. |  | | |  |  |  | |
| **Horizontal width** |  |  |  |  |  |  |  |  |  | |  |  |  | |
| \|Ac-Ac\| (%\|^a^) | Nasal width | 44.55 | 3.21 | 45.73 | 3.47 | 45.50 | 2.83 | 0.23 |  | | 1.49 |  | 0.03 | |
| \|Zy-Zy\| (%\|^a^) | Zygomatic width | 152.91 | 6.83 | 158.05 | 7.29 | 155.93 | 7.15 | 0.01 | * | | 5.09 |  | 0.09 | |
| \|Ch-Ch\| (%\|^a^) | Mouth width | 54.10 | 4.89 | 55.30 | 4.28 | 55.47 | 4.75 | 0.38 |  | | 0.98 |  | 0.02 | |
| \|Go-Go\| (%\|^a^) | Gonial width | 120.81 | 8.49 | 126.50 | 9.20 | 126.02 | 8.40 | 0.01 | * | | 4.97 |  | 0.09 | |
| \|Go-Go\|/\|Zy-Zy\| | Gonial to zygomatic width ratio | 0.79 | 0.43 | 0.80 | 0.42 | 0.80 | 0.38 | 0.24 |  | | 1.46 |  | 0.03 | |
| **Vertical height** |  |  |  |  |  |  |  |  |  | |  |  |  | |
| \|N-En\| (%\|^a^) | Vertical position of nasion to eyes | 6.19 | 1.90 | 4.73 | 2.52 | 6.35 | 2.24 | 0.00 | * | | 5.73 |  | 0.10 | |
| \|N-Sn\| (%\|^a^) | Nasal length | 57.39 | 3.03 | 57.35 | 3.99 | 57.56 | 3.64 | 0.97 |  | | 0.03 |  | 0.00 | |
| \|N-Zy\| (%\|^a^) | Vertical position of cheek | 36.71 | 7.05 | 36.98 | 5.49 | 39.32 | 6.49 | 0.18 |  | | 1.75 |  | 0.03 | |
| \|N-Prn\| (%\|^a^) | Vertical position of nasal tip | 40.90 | 2.68 | 40.75 | 3.93 | 41.04 | 2.98 | 0.93 |  | | 0.07 |  | 0.00 | |
| \|N-Ls\| (%\|^a^) | Vertical position of the upper lip | 72.14 | 4.36 | 71.29 | 4.73 | 72.29 | 4.64 | 0.61 |  | | 0.49 |  | 0.01 | |
| \|Sto-Gn\| (%\|^a^) | Mandibular height | 52.27 | 4.30 | 52.92 | 4.95 | 55.24 | 4.13 | 0.02 | * | | 4.31 |  | 0.08 | |
| \|Ls-Li\| (%\|^a^) | Lip vermilion thickness | 17.83 | 2.68 | 17.19 | 2.76 | 17.07 | 2.32 | 0.40 |  | | 0.93 |  | 0.02 | |
| \|Gla-Sn\|/\|Sn-Gn\| | Upper to lower face height ratio | 1.11 | 0.09 | 1.13 | 0.13 | 1.06 | 0.10 | 0.02 | * | | 4.00 |  | 0.07 | |
| \|N-Gn\|/\|Zy-Zy\| | Height to width ratio of the face | 0.87 | 0.04 | 0.84 | 0.05 | 0.87 | 0.05 | 0.02 | * | | 6.38 |  | 0.10 | |
| \|Gla-ls\|/\|Zy-Zy\| | Height to width ratio of the mid-face | 0.53 | 0.30 | 0.50 | 0.31 | 0.52 | 0.31 | 0.00 | * | | 6.38 |  | 0.11 | |
| \|Ps-Pi\| (%^a^) | Eye height | 14.67 | 1.46 | 14.40 | 1.40 | 13.44 | 1.67 | 0.00 | * | | 6.48 |  | 0.11 | |

* *p* < 0.05 (*p* values were adjusted for multiple comparisons using the Benjamini-Hochberg method.^1^)

a, percentage to the distance between right Ex and left Ex.

† For detailed definitions see Tanikawa et al. (2016) ^4^.

S.D., standard deviation.

***Supplementary Table S8. Comparisons of three subject groups (Codes 1–3) for body composition parameter***

|  | Code 1  Balanced- and low-calorie intake group  (n = 45) | | Code 2  High-calorie intake group  (n = 36) | | | Code 3  Imbalanced- and low-calorie intake group (n = 34) | | | p-value | | F-value | | Effect size (ɳ^2^) | | |
| --- | --- | --- | --- | --- | --- | --- | --- | --- | --- | --- | --- | --- | --- | --- | --- |
|  | Mean | S.D. | | Mean | S.D. | | Mean | S.D. |  | | |  | |  |  |
| Body weight (kg) | 49.6 | 0.7 | | 52.0 | 0.7 | | 51.2 | 0.8 | 0.07 | NS | | 2.7 | | 0.05 |  |
| Total skeletal muscle mass (kg) | 20.2 | 0.3 | | 20.5 | 0.3 | | 20.1 | 0.4 | 0.67 | NS | | 0.4 | | 0.01 |  |
| Total body fat mass (kg) | 12.2 | 0.4 | | 14.0 | 0.4 | | 13.7 | 0.5 | 0.01 | ** | | 5.2 | | 0.08 |  |
| Body fat percentage (%) | 24.8 | 4.1 | | 25.7 | 3.5 | | 27.7 | 4.3 | 0.006 | ** | | 5.3 | | 0.09 |  |
| Body mass index (BMI)  (Kg/m^2^) | 19.8 | 0.2 | | 20.8 | 0.3 | | 20.4 | 0.3 | 0.02 | * | | 3.9 | | 0.07 |  |
| Fat free mass index (FFMI^5^) (Kg/m2) | 14.8 | 1.0 | | 15.4 | 1.2 | | 14.9 | 1.0 | 0.02 | * | | 3.9 | | 0.06 |  |
| Fat mass index (FMI^5^) (Kg/m2) | 4.9 | 1.1 | | 5.3 | 1.0 | | 5.8 | 1.3 | 0.004 | ** | | 5.8 | | 0.09 |  |

**<0.01, * *p* < 0.05

ANOVA, analysis of variance; S.D., standard deviation.

***Supplementary* *Table S9. Comparisons of three subject groups (Codes 1–3) for health history variables*** ***in the 43 randomly selected participant).***

| Variable | Code 1  (n =20) | | Code 2  (n =12) | | Code 3  (n =11) | | p-value | | F-value | Effect size (ɳ^2^) |
| --- | --- | --- | --- | --- | --- | --- | --- | --- | --- | --- |
|  | Mean | S.D. | Mean | S.D. | Mean | S.D. |  | |  |  |
| Birth weight (g)^a^ | 3041 | 331 | 3057 | 407 | 2965 | 508 | 0.859 |  | 0.152 | 0.01 |
| Two year change in weight (Kg)^b^ | 0.85 | 3.01 | 3.33 | 4.00 | 3.40 | 3.75 | 0.079 |  | 2.712 | 0.12 |
| Physical activity level | 1.95 | 0.23 | 2.00 | 0.43 | 1.73 | 0.47 | 0.169 |  | 1.862 | 0.09 |
| Duration of physical exercises (hours/week) | 3.55 | 3.15 | 3.08 | 3.26 | 3.64 | 3.01 | 0.896 |  | 0.111 | 0.01 |
| Sleep duration (hours/day)^b^ | 6.22 | 0.77 | 5.88 | 0.96 | 5.70 | 1.06 | 0.283 |  | 1.304 | 0.06 |
| Menarche age (years old) | 11.60 | 1.93 | 11.17 | 1.34 | 11.27 | 1.49 | 0.750 |  | 0.289 | 0.01 |

1. Two samples in Code 2 and two samples in Code 3 were excluded from this calculation due to unanswered data
2. One sample in Code 3 was excluded from this calculation due to unanswered data
3. One sample in Code 1 was excluded from this calculation due to unanswered data

***Supplementary Table S10. Definitions of the soft tissue landmarks on the facial 3D images***

| Landmark |  | Definition | Single | Paired |
| --- | --- | --- | --- | --- |
| Glabella | Gla | The most prominent midpoint between the eyebrows (Mathematically defined) | ✓ |  |
| Nasion | N | The deepest point on the nasofrontal region in a sagittal plane | ✓ |  |
| Exocanthion | Ex | The point at the outer commissure of the eye fissure |  | ✓ |
| Endocanthion | En | The point at the inner commissure of the eye fissure |  | ✓ |
| Palpebrale superius | Ps | The highest point on the middle portion of the free margin of each upper eyelid |  | ✓ |
| Palpebrale inferius | Pi | The lowest point on the middle portion of the free margin of each lower eyelid |  | ✓ |
| Porion | Po | The most superior point on each ear canal |  | ✓ |
| Zygomaticus | Zy | The most prominent point on the zygomatic area from the 45° oblique view (Mathematically defined) |  | ✓ |
| Pronasale | Prn | The most protruding point of the apex nasi in a sagittal plane | ✓ |  |
| Alar curvature point | Ac | The most posterolateral point in the curved base line of the alar, indicating the facial insertion of the base of the nasal wing base onto the curvature of the base |  | ✓ |
| Subnasale | Sn | The midpoint of the base of the columella where the lower border of the nasal septum meets the surface of the upper lip | ✓ |  |
| Labiale superius | Ls | The outermost point on the mucocutaneous border of the upper lip in the mid-sagittal plane | ✓ |  |
| Cheilion | Ch | The outer corner of the mouth where the outer edges of the upper and lower vermilions meet |  | ✓ |
| Labiale inferius | Li | The outermost point on the mucocutaneous border of the lower lip in the mid-sagittal plane | ✓ |  |
| Gnathion | Gn | The most anterior inferior point of the chin in the mid-sagittal plane (Mathematically defined; see Tanikawa et al.^4^) | ✓ |  |
| Zygomaticus | Zy | The most lateral point on the facial outline (Mathematically defined; see Tanikawa et al.^4^) |  | ✓ |
| Gonion | Go | The most inferior and lateral point on the external angle of the mandible (Mathematically defined; see Tanikawa et al.^4^) |  | ✓ |

***Supplementary Fig S1.*** ***Coordinate system and landmarks employed in the present study.*** Please see a reference ^4^ for details.


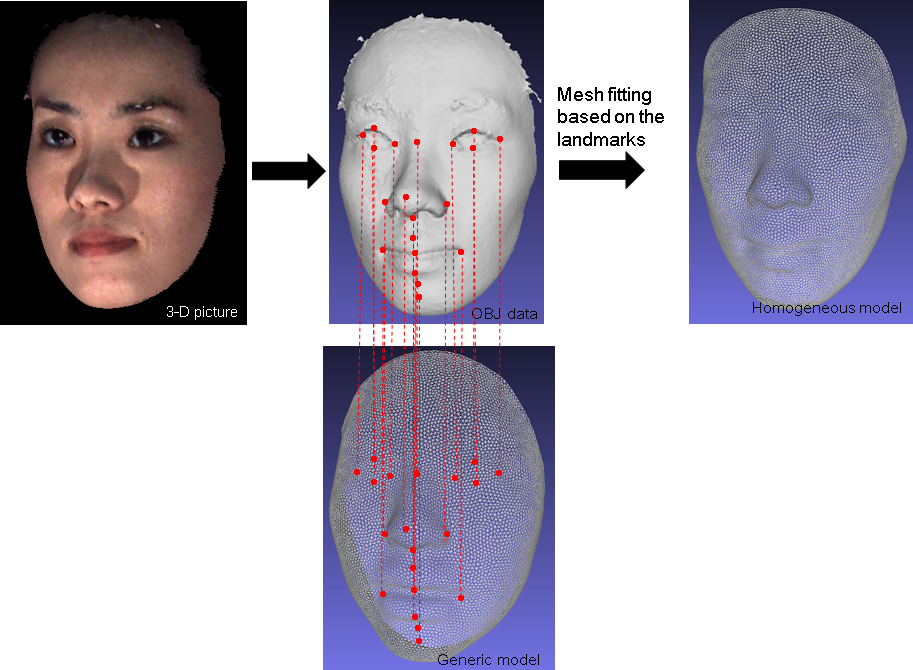


***Supplementary Fig S2. Schematic illustration of the wire mesh fitting and the point cloud of the face that were examined ^5^.***

For each facial surface (top left), fitting of high-resolution template meshes or a generic model (bottom center) was performed using commercial software (HBM-Rugle, Medic Engineering Co., Kyoto) based on the landmarks assigned to each 3D image (top center). This method automatically generated a homogeneous model (top right) that consisted of 6,017 points (i.e., fitted mesh or semi-landmark nodes) on the wire mesh for each model with landmark anchors. The semi-landmark nodes on the wire mesh of the homogeneous model generated for each face were used for further calculation.

**References**

1 Benjamini, Y. & Hochberg, Y. Controlling the False Discovery Rate - a Practical and Powerful Approach to Multiple Testing. *J R Stat Soc B* **57**, 289-300, doi:DOI 10.1111/j.2517-6161.1995.tb02031.x (1995).

2 Hennekam, R. C. *et al.* Elements of morphology: standard terminology for the nose and philtrum. *Am J Med Genet A* **149a**, 61-76, doi:10.1002/ajmg.a.32600 (2009).

3 Chao, J. W., Lee, J. C., Chang, M. M. & Kwan, E. Alloplastic Augmentation of the Asian Face: A Review of 215 Patients. *Aesthetic Surgery Journal* **36**, 861-868, doi:10.1093/asj/sjw013 (2016).

4 Tanikawa, C., Zere, E. & Takada, K. Sexual dimorphism in the facial morphology of adult humans: A three-dimensional analysis. *Homo* **67**, 23-49, doi:10.1016/j.jchb.2015.10.001 (2016).

5 Hattori, K., Tatsumi, N. & Tanaka, S. Assessment of body composition by using a new chart method. *Am J Hum Biol* **9**, 573-578, doi:10.1002/(sici)1520-6300(1997)9:5<573::Aid-ajhb5>3.0.Co;2-v (1997).

6 Tanikawa, C., Akcam, M. O. & Takada, K. Quantifying faces three-dimensionally in orthodontic practice. *J Cranio Maxill Surg* **47**, 867-875, doi:10.1016/j.jcms.2019.02.012 (2019).
